# On the Cementitious Mixtures Reinforced with Waste Polyethylene Terephthalate

**DOI:** 10.3390/ma17215351

**Published:** 2024-10-31

**Authors:** Cristiano Giuseppe Coviello, Armando La Scala, Maria Francesca Sabbà, Leonarda Carnimeo

**Affiliations:** 1 Department of Architecture, Construction and Design, Polytechnic University of Bari, Via Orabona 4, 70125 Bari, Italy; c.coviello@phd.poliba.it (C.G.C.); armando.lascala@poliba.it (A.L.S.); 2 Department of Electrical and Information Engineering, Polytechnic University of Bari, Via Orabona 4, 70125 Bari, Italy; leonarda.carnimeo@poliba.it

**Keywords:** recycling, PET-reinforced cementitious mixtures, PET fibers and aggregates, strengthened mechanical properties, mortar, concrete

## Abstract

The last decade was dominated by a serious problem that now affects all the planet’s natural ecosystems: the increasing growth of plastics and microplastics that are difficult to dispose of. One strategy to mitigate this problem is to close the life cycle of one of them—polyethylene terephthalate (PET)—by reusing it within the most common building materials, such as mortars and concretes. The reuse of PET waste as aggregates also allows us to limit the CO_2_ emissions released during the production of natural aggregates. This paper analyzes the outcomes of many studies carried out on the characteristics of cementitious mixtures reinforced with waste PET material. Many researchers have demonstrated how PET used as reinforcement of mortars and concretes can produce an increase in the mechanical strengths of the corresponding cementitious mixtures without PET. The tensile strength of this resin is higher than that of concrete; so, by combining the two materials it is possible to obtain a mixture with an overall higher tensile strength, resulting in increased flexural strength and reduced cracking. Using an effective size of PET fibers, it is possible to achieve an increase in the ductility and toughness of the cementitious mixture. Several studies reveal that PET reinforcement reduces the density with a consequent decrease in weight and structural loads, while the workability increases using spherical and smoother PET aggregates.

## 1. Introduction

About 13 million tons of plastic enter our oceans each year, causing damage to biodiversity, economies, and, potentially, to our own health [1]. It is known that 70% of total plastic bottle consumption is discarded as waste without becoming a potential reused resource [2]. This is a problem since PET waste is not biodegradable and is destined to remain in nature for hundreds of years [3,4]. Over the years, civil engineering has also wondered about searching for a useful strategy to mitigate this problem by recovering some of the PET plastic waste put into landfills. With this aim, the idea of using PET as a reinforcement material for common cementitious mixtures developed. Such a solution has proven to be sustainable and in the direction of the circular economy [5]. The hydrolysis of PET is a well-known and applicable phenomenon to break down the resin into its molecular components. However, such processes are difficult to apply on a large scale and on large quantities of material. Reusing PET would be a sustainable and cost-effective solution [6]. In fact, using recycled PET aggregate in concrete mixtures instead of the natural virgin aggregate would reduce the CO_2_ emissions associated with the production of new material [7].

Although cementitious compounds are the most widely used materials in the construction industry [8], they are also the most polluting to the environment. In fact, these materials require the use of non-renewable resources (such as fine and coarse natural aggregates or fossil fuels for energy production), which will risk being depleted for future generations if continued to be extracted [9]. Added to this factor is the deterioration of the natural environment (flora and fauna) caused by the extraction phases of raw materials [10]. In addition, the subsequent processing phases of virgin raw materials produce the release of dust and toxic substances such as greenhouse gases that pollute the environment, exacerbating the problem of climate change [11,12]. Nowadays, the substitution of fine and coarse natural aggregate with recycled PET inside concrete and mortar mixtures is considered a viable solution.

Typically, PET is easy to find in a mass-consumption item such as plastic bottles of any kind. From them, minor elements can be shredded in the form of fibers or aggregates to be later introduced within cement mixtures [13,14]. To prevent other materials from interfering in the interaction between PET and the cement matrix, firstly it is necessary to clean the surfaces of the plastic elements by removing any non-polymeric material [15,16]. After that, through specific proportions by weight or volume, PET reinforcing elements can be introduced in the cement matrix in various shapes. In this way, several researchers [17,18,19] proved that coupling a material with a higher tensile strength and a lower weight, compared with those typical of concrete [20] and construction mortars [21], can result in a mixture with higher flexural strength [22,23] and lower structural weight [24], respectively.

In Table 1, a comparison of the mechanical properties of virgin and recycled PET fibers is shown. The process of converting PET fibers from virgin to recycled was performed by an extruder, in which in the spinning stage, the fibers are stretched through three sets of five rollers. This operation enhances molecular orientation and crystallinity, so tensile strength also tends to improve [25].

The introduction of PET waste material also produces a great advantage in reducing chloride permeability [30]. This is extremely beneficial for reinforced concrete structures where it is necessary to protect steel bars from chloride entrance.

Rahimi [31] discovered that by increasing the percentage of PET within the concrete, an increasing reduction in sulfuric acid erosion over time could be achieved, improving its durability [32]. All these aspects are just a part of many other improvements that the use of PET plastics can generate in cementitious mixtures.

**Figure 1:**
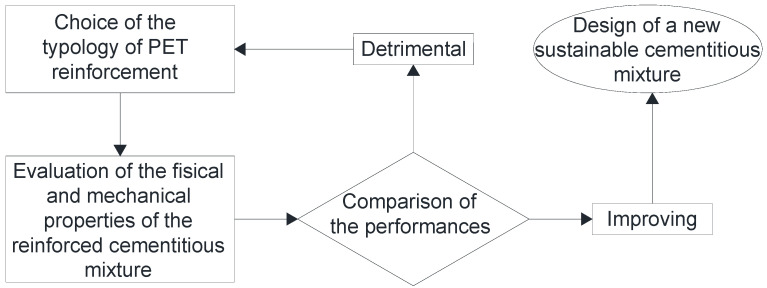
Overview flow-chart.

In this review, the reuse of PET in the form of resin, aggregate or fiber (FRC) [33,34], is presented. Also, the criteria for adding these reinforcing elements are explained. It will be focused on the geometric and shape characteristics of PET fibers and aggregates, demonstrating the importance of these dimensional aspects. Finally, the contributions that researchers over the past 15–20 years have given to the topic at hand are presented. In particular, emphasis will be placed on the correspondence between the PET reinforcement element and the improvement of properties in the fresh and hardened state of mortars and concretes reinforced with increasing percentages of PET. Figure 1 shows the methodological process followed for the right design of the PET reinforcement of cementitious mixtures.

## 2. Research Justification

The emergence of the research discussed in this paper is directly related to the most urgent problem of the 20th century: CO_2_ emissions. Cement production on average produces between 5% and 8% of global CO_2_ emissions [35,36]. Generally, it can be assumed that every kilogram of Portland cement produced generates 0.81 kg of CO_2_, and as a result, approximately 2.1 billion tons of CO_2_ are produced each year [37]. In cement production, the steps to be considered for the correct evaluation of CO_2_ consumption are the following: emissions from the combustion of fossil fuels in the clicker production process to heat the limestone raw materials (CaCO_3_) [38] at a temperature of 1450 °C; emissions from the calcination process in which the raw material is converted into cement; indirect emissions from the transport and delivery of raw materials and finished products; emissions from fossil fuels that provide electricity generation for the efficient use of machines and equipment [39,40].

About the production of natural aggregates of concrete, In Energy reports that the CO_2_ emissions for the extraction and processing phases of 1 ton of fine and coarse aggregates is on average 8.1 kg [41]. This value takes into account the different stages involved in transforming a raw material into a finished product that can be used in the construction industry, quarrying, onsite transportation, crushing, sieving and sorting, and at the end, transportation to the construction site (50 km).

In the concrete industry, 90% of CO_2_ is emitted from the production of cement alone [42]; replacing natural aggregates with recycled PET ones would significantly reduce the environmental impact. A recycled aggregate, in contrast to a virgin one, would certainly allow an initial reduction of CO_2_ associated with the production of the material ex novo. The use of recycled PET can lead to lower costs compared to natural aggregates in certain circumstances, helping to reduce production costs and improving the profitability of the activities that employ it. Furthermore, the adoption of recycled materials can help to raise consumer and business awareness of the importance of recycling and waste reduction, promoting greater environmental responsibility. In this chapter, a simplified evaluation of the CO_2_ reduction that would be achieved by using recycled PET aggregates instead of natural ones was carried out. As in recent years, construction companies are focusing heavily on CO_2_ savings; it is useful to provide a practical and numerical approach to the concrete benefits of this research.

A justification on real cases is considered a guide for construction companies that are looking for a sustainable and less polluting solution.

To perform a correct estimation of the energy and environmental emission savings, it would be necessary to evaluate the entire life cycle of the aggregate itself. In particular, the factors that should be considered should be as follows:

- The production consumption of natural and recycled PET plastic aggregates;
- The consumption associated with the transport of the aggregates to the site of use;
- The durability of the natural and plastic aggregates.

In the overall calculation for estimating the global CO_2_ consumption produced by a reinforced concrete building, it is important to also consider the CO_2_ emissions associated with processes concerning water. In fact, during the cement production process, water is used for the hydration of clinker Portland which releases CO_2_ as a by-product. To ensure proper workability during casting, concrete requires water, which is an expense in terms of CO_2_ due to transportation from the company to the construction site by vehicles burning fossil fuels. Water extraction and treatment can cause environmental impacts such as lowered groundwater levels or water contamination, which in turn may require the use of additional resources and cause CO_2_ emissions during mitigation activities. Considering these factors, the CO_2_ consumption that occurs in the water usage phases is 0.000249 kg per ton of water [43].

The extremely energy-intensive and polluting steps associated with cement production in concrete could be partially replaced by the hydrolysis processes of PET used as a resin to replace common cements.

In addition, the high light weight of PET would allow an important reduction in CO_2_ emissions due to a reduction in the load traveling through the transport means from the production plant to the construction site. Less weight corresponds to a reduction in fuel and thus, a reduction in CO_2_ emitted in the combustion phase. From a thermal point of view, PET is an excellent thermal insulator; therefore, the application of cementitious mixtures would promote better thermal insulation of a building’s structure by reducing the need to adopt very energy-intensive external heating systems. In addition, PET is an easily recyclable material; reusing it in new cementitious mixtures would reduce CO_2_ emissions associated with landfill incineration steps. Based on these considerations, it is possible to state that PET saves a great amount of energy and fossil fuels in favor of less CO_2_ released into the environment.

According to the studies [43], the use of waste PET in concrete reduces energy by 60 MJ/kg, which corresponds to a CO_2_ reduction of about 3.38 kg per kg of recycled PET.

**Figure 2:**
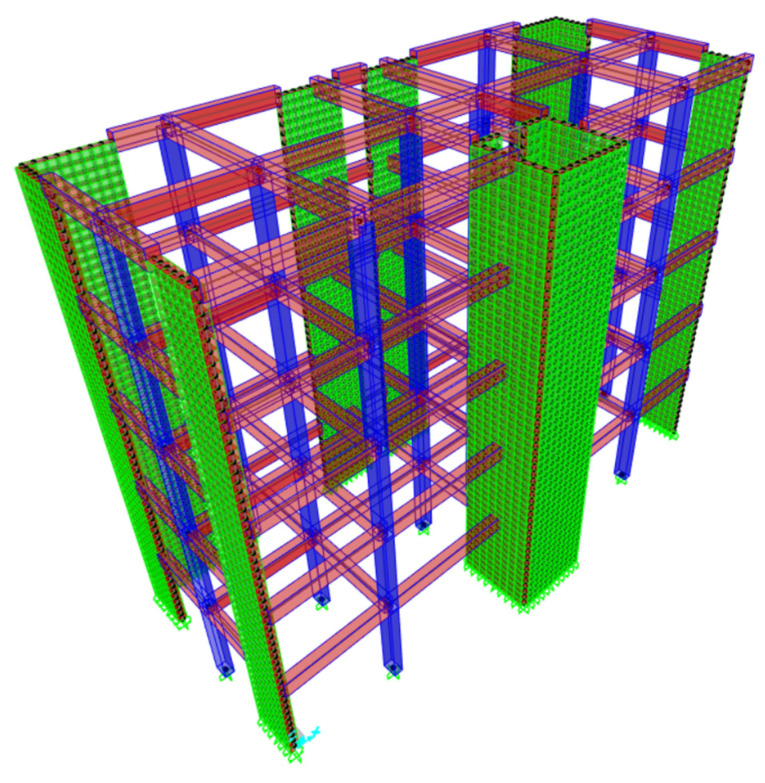
Example of a reinforced concrete structure with 10 columns, 26 beams, and 11 load-bearing walls of 5 floors.

Therefore, the example of a reinforced concrete building first made with traditional conglomerate and then with PET-reinforced conglomerate in the form of resin or aggregate has been considered below. Table 2 presents the CO_2_ values that were considered in the calculation of the overall computation for the total volume of the considered building, while Figure 2 shows the reinforced concrete building that was computed. The reinforced concrete structure has five floors. The columns have uniform sections of 30 cm × 50 cm, the beams have sections 30 cm × 50 cm, and the bearing walls are 30 cm thick. The total volume of concrete used in the construction is 179.55 m^3^. Considering the different application types that PET can assume in concrete mixtures, the four scenarios described in Table 3 were contemplated. After calculating the total CO_2_ consumption in the four scenarios, the values were compared to numerically show the CO_2_ savings that would be achieved by using PET in concrete.

The value of the replacement ratio of 5% by weight was chosen because in Rahmani and Asadi [45,46], they showed that using this percentage, the compressive strength of concrete increased between 5% and 11%. As can be seen from Table 3, the highest CO_2_ emissions in tons occur for scenario 0, in which PET is not included. In contrast, in scenarios 1, 2, and 3, the 5% substitution by weight of PET produces a significant decrease in CO_2_ emissions. Specifically, in scenario 1, PET is used as a partial replacement of traditional Portland cement, while in scenario 2, it is used as a replacement for fine natural aggregates. In scenario 3, the assumptions of the previous two scenarios are combined by considering 2.5% by weight of PET as a substitute for cement and natural aggregates, respectively.

The results show that scenario 2 is the most cost-effective in terms of CO_2_ emission reduction for a reinforced concrete structure. In fact, in this case, the overall CO_2_ reduction result was 31.02%, while for scenario 1 and 3, the results were 23.62% and 27.32%, respectively. Therefore, although concrete is the component that emits the largest amount of CO_2_ in 1 m^3^ of concrete, the most cost-effective solution to reduce pollutant emissions is to replace natural fine aggregates with PET reinforcement. This depends on the fact that fine aggregates have a much higher mass in the unit volume of the mix than Portland cement. This occurs because fine aggregates have a mass in the unit volume of the mix that is about two times higher than that of Portland cement.

Through the comparison of these three scenarios, it was possible to analytically demonstrate how the use of PET in concrete can become a resource for solving the problem of the environmental impact.

In view of the advantages associated with the use of PET in cement mixes, it felt appropriate to construct a roadmap that illustrates how this material can be applied integrally within a construction company. The steps for using PET must be planned according to the type of physical or mechanical properties of the mix that should be improved. Based on the end goal of the company, it is necessary to specifically plan the steps that precede the manufacture of the mixture. Operationally, in the first instance, a company should ensure the recovery of the waste material by extracting it from a specific site [47]. After recovery, an initial selection of the type of PET to be used should be made, i.e., the cap, the label, and the stiffest part of the bottom of the bottle could be removed. The selected parts must be cleaned and treated to remove materials different from PET and to wash the plastic surfaces that will be used in the next phases [48]. Depending on the property to be improved, the treated PET must be cut according to specific geometries. Fiber may have an improving impact on flexural strength at the cost of compressive strength and workability of the cement mix. A regular fine aggregate may improve compressive strength at the cost of flexural strength. Cutting must be carried out according to fixed geometries that depend on the size of the larger aggregate that will be introduced into the cement mix. For better adhesion between the plastic and matrix, it is recommended to use a fiber with a length greater than twice the diameter of the larger aggregate. To ensure that the mechanical properties of the PET are maintained even after the treatments, periodic tests must be carried out to assess the tensile strength, the Young’s modulus, and the breaking strain of the material. The concrete or the mortar mixture to be reinforced is then designed. In it, a specific quantity by volume or weight of fibers or plastic aggregates to be used will be defined. The mixture then needs to be poured into cylindrical (150 mm long and 300 mm in diameter) and prismatic (600 × 150 × 150 mm or 500 × 100 × 100 mm) molds [49]. After hardening, the compressive and flexural strength values must be measured at 28 days. The purpose of this first measurement is to assess the benefit of the introduction of the PET reinforcement. In addition to the strength properties, the properties in the fresh state, as workability and air content, must also be evaluated. Only once a sufficient number of specimens have been tested, the mix design can be considered reliable and can be used within the production of the company.

## 3. Potential Applications of PET for Reinforcing Cementitious Mixtures

### 3.1. PET Aggregates

The first type of PET reinforcement is in its aggregate form, where this material assumes the same role as that of the natural aggregate in concrete or in the mortar mixture. The dimensions of the particles used in mortar mixtures are in the range of 0.25 mm and 4 mm [33]. Ferreira et al. [50] compared the behavior of two different lamellar and irregularly shaped PET aggregates with other regular and cylindrical granulated PET aggregates. The same cylindrical shape was studied by Abed et al. [51] where PET aggregates, with dimensions smaller than 4.75 mm, were used as reinforcement in mortars. Saikia [52] compared the mechanical behavior of three different PET aggregates (angular and irregular particles, both fine and coarse, and spherical/cylindrical heat-treated pellet-shaped products) in nine concrete mixtures. Similarly, other researchers have studied the behavior of PET aggregates with different geometry, size, and percentage replacement by weight or volume in mortars or concretes (Table 4).

**Figure 3:**
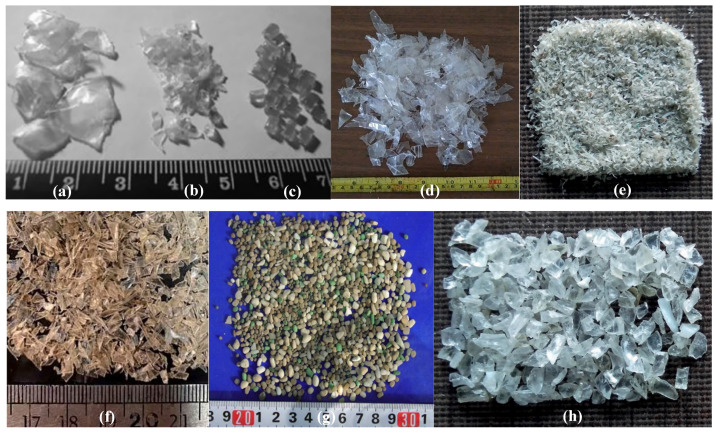
Types of PET aggregates used in some research: (**a**) lamellar and irregular larger than (**b**); (**c**) regular and cylindrical [50]; (**d**) shredded irregular shape [53]; (**e**) 300–150 μm flaky shape [54]; (**f**) angular shape [55]; (**g**) smooth sphere shape [56]; and (**h**) 2.36–1.18 mm flaky shape [54].

All the shapes of PET aggregates that have been used in previous research are presented in the Figure 3.

In several studies, to be able to identify the sizes of the PET particles introduced into the mixture, a grading test of the replaced aggregates [53] is performed and the particle size curve is drawn [57]. The sieve analysis is even more truthful if the plastic particles are similar in shape to the replaced natural aggregate [58]. In fact, similar shapes between PET and natural aggregates will ensure more similar behavior.

**Table 4:** Some of the most common PET aggregates used in the literature.

Years	Researchers	Matrix	Geometry	Size (mm)	Replacement (%)
2009	Albano et al. [59]	Concrete	Lamellar and irregular	Small: 2.6; big: 11.4	10; 20 in volume
2010	Hannawi et al. [60]	Mortar	Flaky and irregular	1.6–10	3; 10; 20; 50 in volume
2013	Rahmani et al. [45]	Concrete	Lamellar and irregular	0.15–7	5; 10; 15
2014	da Silva et al. [61]	Mortar	Pellets and flakes	1–4	5; 10; 15 in volume
2015	Araghi et al. [62]	Concrete	Lamellar and irregular	0.15–4.75	5; 10; 15 in volume
2016	Islam et al. [63]	Concrete	Flaky and round shape	2.5–40	20; 30; 40; 50 in volume
2019	Perera et al. [64,65,66]	Concrete	Flaky	0.075–26	3; 5
2019	Lee et al. [67]	Concrete	Flaky	1–13	10; 20; 30 in volume
2020	Sposito et al. [68]	Mortar	Granular	0.1–4.5	2.5; 5; 10; 15; 20 in volume
2021	Foti and Lerna [69]	Mortar	Granular	2	0; 25; 50; 75 in volume
2022	Khan et al. [70]	Mortar	Powder	0.075–0.6	2.5; 5; 7.5; 10 in volume
2023	Coviello et al. [5]	Screed	Flaky and irregular	0.5–5	1; 2; 3 in weight

### 3.2. PET Resin

Another successful application of PET reinforcement is as a resin [71]. In this case it is necessary to perform a preliminary depolymerization by glycolysis that splits the plastic material into its monomers and/or oligomers suitable for possible repolymerization [72,73]. In this procedure, the mixture is added into a three-necked glass flask and placed on a heating seal [74]. The set-up of the glycolysis process consists of a three-necked glass flask, a middle-motorized shaker, a thermometer, and a condenser [73]. In [75], the PET resin added in 5%, 10%, and 15% by mass of cement spread the values of the consistency and workability compared with the reference mortar.

The maximum temperature reached by the heater is 210–220 °C and that value is kept until the PET particles in the mixture dissolve [74]. After this process, the waste plastic can have the same role as the binder in the cementitious mix. The main advantages of this second type of reuse of PET in mortars is that the abrasion resistance increases from 10% to 33.50% and the tensile strength reaches a significant enhancement from 8% to 41% because the number of polymer chains is increased. The polymer chains act as the connection that is able to hold the components together and reduce the propagation of cracks [76].

The use of PET in resin form provides excellent advantages within mortars since it can achieve the following: (I) increases the tensile strength; (II) increases the abrasion resistance; (III) increases the compressive strength; and (IV) increases the slump flow. However, these benefits occur only when curing of the cementitious mixtures takes place in air and not in water [76]. In [71], it has been found that for the specimens cured for 420 days, upon increasing PET resin content from 25% to 35%, the compressive strength, flexural strength, and indirect tensile strength increased by 10.2%, 11.8%, and 9.9%, respectively. Moreover, waste materials have collectively reduced the water absorption.

### 3.3. PET Fibers

The most common use of PET reinforcement within cementitious composites is in the form of fibers in which the aspect ratio (AR) of one dimension to the other exceeds at least an order of magnitude [77]. Generally, the methods of PET fiber production are hand-cutting plastic bottles or using shredding machines; the first method can be used for small amounts of material, while the second for more industrial and mass use. The affordability of the use of recycled PET fibers in an industrial process depends on the amount of recycled PET fiber as a function of time and energy consumption [78]. The relationship with all evaluation parameters and the operating speed of the machine is linear [79]. Therefore, a machine that can shred PET fiber should be designed considering the specific needs of a company [80]. In [81], the final production cost of a PET shredding machine is $191, which is relatively affordable for local recyclers.

The scientific literature, over the years, has presented several experimental case studies in which PET reinforcement material has been applied using elements with different sizes and geometries. R.P. Borg et al. [82], for example, examined the behavior of mortars reinforced with two different PET fibers that were 50 mm and 30 mm long.

**Figure 4:**
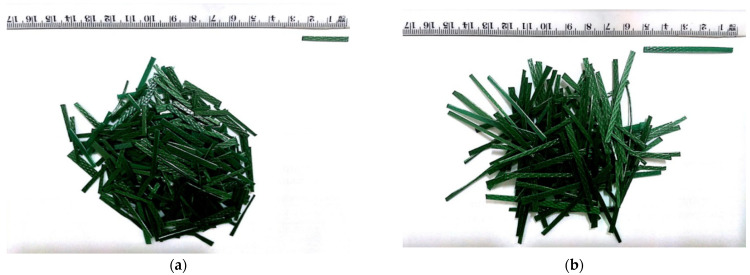
PET fiber examples of width 20 mm, thickness 0.5 mm, length 50 mm (**a**), and length 50 mm (**b**) [95].

Table 5 collects the landmark research on the use of PET fibers in concrete mixes in chronological order. In Figure 4, an example is presented of PET fiber reinforcement, where the dimensions and the geometry of the elements are always the same in (a) and (b), respectively. The surface of the illustrated fibers is not linear but has asperities to improve the grip between the fiber and matrix.

**Figure 5:**
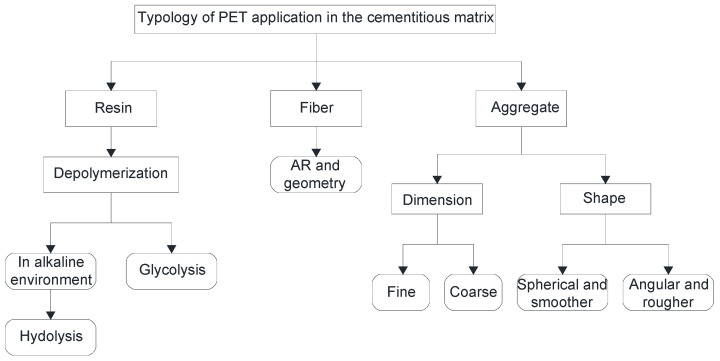
Screening of the different types of PET reinforcement in the cementitious matrix.

Figure 5 shows a schematic flow chart in which the potential applications of PET reinforcement of cementitious mixtures are summarized.

**Table 5:** Some of the most common PET fibers used in the literature.

Years	Researchers	Matrix	Thickness (mm)	Width (mm)	Length (mm)	Replacement (%)
2010	Kim et al. [83]	Concrete	0.2	1.3	50	10; 20 in volume
2011	Foti [84]	Mortar	-	5	Lamellar: 32; 35. Circular: 30; 50; 60	3; 10; 20; 50 in volume
2011	Oliveira and Castro-Gomes [34]	Concrete	0.5	2	35	5; 10; 15
2014	Fraternali et al. [85]	Mortar	(a) 1.10; (b) 0.7	Circular fibers	(a) 40; (b) 52	5; 10; 15 in volume
2016	Corinaldesi et al. [86]	Concrete	0.2	1.2	40	5; 10; 15 in volume
2017	Fernandez et al. [87]	Concrete	0.23; 0.29; 0.41; 0.48	1; 1; - 1	6	20; 30; 40; 50 in volume
2018	Poonyakan et al. [88]	Concrete	<0.3	Equivalent diameter	12–65	3; 5
2018	Shahidan et al. [89]	Concrete	-	5	50	10; 20; 30 in volume
2019	Alani et al. [90]	Mortar	0.3	3.5	40	2.5; 5; 10; 15; 20 in volume
2020	Mohammed et al. [91,92]	Mortar	0.4	1.2	20; 40	25; 50; 75 in volume
2023	Sabireen et al. [93]	Mortar	-	1.5–3	75–100	2.5; 5; 7.5; 10 in volume
2024	Parhi and Patro [94]	Concrete	0.105	5	35	0.3; 0.4; 0.5 in volume

## 4. PET-Reinforced Mixture Design

### 4.1. Substitution Criteria

In the design and study of new reinforcement materials for cementitious mixes, the replacement of organic aggregates can be partial or total depending on the intended use of the plastic materials. In fact, the function could be as reinforcement or as a total replacement of the pre-existing matrix aggregates. Coviello et al. [5] prepared screeds by mixing white sand with Portland cement and flaky and irregular PET aggregates. Akçaözoğlu et al. [33] compared the behavior of the mortar mixtures M1 and M2, that contain 25.64% in weight of PET without organic sand aggregate, with that of the mortar mixtures M3 and M4, that contain both PET and sand at 16.95% and 33.90% in weight, respectively.

Different to other research, in which a certain percentage by weight or by volume of PET is added, in [33], the partial or total replacement of natural aggregates with polyethylene terephthalate results in a more sustainable solution. In fact, the reduction in the use of natural aggregates decreases the demand for a non-renewable resource, and consequently, the costs relating to extraction and producing, without considering the advantages in terms of environmental sustainability.

The criterion of substitution according to a volume-equivalent quantity requires that the particle size of the natural aggregates to be substituted be determined preliminarily. Saikia [52], for each class of concrete mix containing PET aggregate, prepared three sub-classes by replacing 5%, 10%, and 15% in volume of natural aggregates by an equal volume of each type of PET aggregate. Hannawi et al. [60] analyzed the physical and mechanical properties of mortars containing 3%, 10%, 20%, and 50% addition of PET plastic as sand replacement in volume, as shown in Table 4. In [96], the slump and the mechanical strengths of nine groups of steel-waste PET fiber reinforced mixtures were measured. In this case, the geometry of the steel and PET fibers were of 28 mm and 30 mm, respectively, while the substitution percentages were 0.5, 0.75, and 1 for the PET fiber.

To achieve the required volume replacement ratio, it is advisable to compare the particle size curve obtained from sieving the PET particles with that obtained from sieving the natural aggregate that has to be replaced [4,55,57]. In this way, each natural aggregate can be substituted by the corresponding PET aggregate having the same size.

The less common criterion for replacing natural aggregates or supplementing them with PET aggregates is usually by weight proportion [77]. Abed et al. [51] has used five different waste PET weight fractions of 0%, 5%, 15%, 25%, and 50% to study the behavior of cement-based reinforced mortar. Sadrmomtazi et al. [57] studied concretes with PET fine particle replacement ratios of 5%, 10%, and 15% by weight of the corresponding substituted natural aggregate.

### 4.2. Aspect Ratio and Geometries of PET Fibers

The effectiveness of the use of the plastic material depends on different factors, e.g., the bond between PET and the matrix made of cement, water, and aggregates. This relates to two aspects: the geometry [97] and the amount of the PET reinforcement used in the mix [98]. Regarding the geometry, the reinforcement can be added using different shapes: particles, square-shaped flakes or strips [99]. For each shape type of PET, partly because of the difficulty in controlling the exact position of these fibers within the cementitious material, the random distribution results in the most effective reinforcement, in which the fibers overlap and intersect themselves without remaining isolated [100].

**Figure 6:**
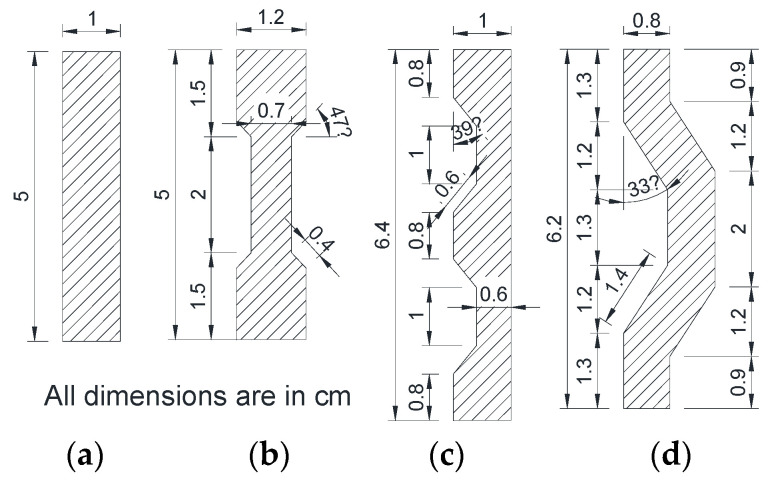
Fiber dimensions of different geometries: (**a**) straight slit sheet, (**b**) flattened end slit, (**c**) deformed slit sheet, and (**d**) crimped end sheet [101].

Marthong and Sarma, in [101], compared the influence of different PET fiber geometries on the behavior of concrete. Their research affirms that the mechanical properties of concrete mixtures progressively increase with the use of straight slit sheet PET fiber, flattened end slit sheet fiber, deformed slit sheet fiber, and crimped end sheet fiber in a volume percentage of 0.5%. Figure 6 shows the differences in geometry and in the dimensions (expressed in cm) of the fibers introduced in the reinforced concrete mixtures.

To improve the performance of cementitious mixtures, the dimensions (length, width, and thickness) of the PET reinforcements taken individually are not as important as the ratio between them. In other words, the factor that most influences the effectiveness of PET reinforcement is the difference between its three dimensions. For this reason, the parameter most commonly used to evaluate an effective interaction between the PET fiber and cement mixture is the aspect ratio (AR). In [102], test results showed that the higher development in strength occurs for AR equal to 33. Similarly, Oliveira and Castro-Gomes [34] found that the use of PET fibers with an AR value of 31 leads to the increase in mechanical performances. Exceeding this average AR value results in an increasing reduction in the mechanical properties of reinforced composites [103,104]. According to [105], AR must be greater than 40 to achieve better mechanical performance.

Different formulations can be used to calculate the AR of fibers with quadrangular cross-sections, generally starting from an equivalent fiber with a circular cross-section and its estimated equivalent diameter ($d_{e}$). So, the AR has been calculated as the ratio of the fiber length on the $d_{e}$ [106].

Oliveira and Castro-Gomes have used Equation (1) in [34]:

(1) $$\lambda =\frac{l}{d_{e}}=\frac{l}{2x\sqrt{\frac{A}{\pi }}}=\frac{l}{2x\sqrt{\frac{bxc}{\pi }}}$$

where l is the fiber length in mm, $d_{e}$ is the equivalent diameter, A is the fiber cross-section area in mm^2^, b is the fiber width, and c is the fiber thickness.

In [104], Meza calculated the fiber $d_{e}$ using Equation (2):

(2) $$d_{e}=\sqrt{\frac{4bh}{\pi }}\;\;$$

where $d_{e}$ is the equivalent diameter, b is the fiber width, and h is the fiber thickness.

## 5. Impact of PET on the Physical/Chemical and Mechanical Properties of the Cementitious Mixtures

### 5.1. PET in Alkaline Environment

**Figure 7:**
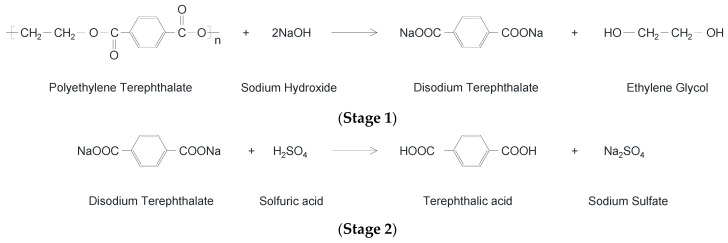
Hydrolysis of PET.

Generally, cementitious mixtures such as those containing Portland cement possess large amounts of calcium silicates and aluminates. These, in the presence of water, produce a strongly alkaline solution of calcium hydroxide (Ca(OH)_2_) in which the pH can reach values of 10–13 [107]. The exposure of PET in an alkaline environment could produce the hydrolysis of PET by breaking down its chemical bonds. As a result, PET would no longer be stable but would suffer a reduction in its mechanical properties. The hydrolysis reaction of PET is described in Figure 7, where PET reacts with two molecules of sodium hydroxide (NaOH) to form disodium terephthalate (Na2TA) and ethylene glycol (EG). Afterwards, disodium terephthalate is neutralized with concentrated sulfuric acid (H_2_SO_4_) and precipitates as terephthalic acid (TA) [108].

Several studies [109,110,111] have stated how, after hydrolysis of PET, its mechanical properties are detrimentally affected. Even so, it has not always been proven that the performance of cementitious mixtures reduces when PET is exposed to an alkaline environment [112]. Negative effects occur when PET possesses geometries that are not appropriate for the conglomerate to be reinforced. Therefore, it is true that PET undergoes a slight reduction in strength after hydrolysis, but within the cementitious mixture, the mechanical parameters measured in the short and long term do not significantly decrease [113].

### 5.2. Influence on the Fresh Properties

The workability of cement mixtures is closely correlated to the shape and quantity of PET reinforcement adopted in the mixture. Regarding the amount and the geometry, the workability is inversely proportional to the ratio of waste addition and the AR [91]. Regarding the shape, angular and rougher aggregates contain the cement mixture’s workability [114], while spherical and smoother aggregates tend to enhance it [50,56]. In [115], for a percentage of substitution in volume of 0.5%, 1%, 1.5%, 2% 2.5%, and 3% of PET fibers of 10 mm length and a 10, 20 aspect ratio, the slump decreased to 7%, 10%, 13%, 16%, 31%, and 40% of the original slump value with 0% plastic fiber content, respectively. One of the causes of reduced workability of PET particles with irregular and sharp shapes is the hydrophobicity of the material [116]. A decrease in workability can also occur because PET particles have a larger specific surface area than sand, causing greater friction between the materials [117]. In [118], melded and shredded fine PET aggregates produced an increase in the workability of concrete mixes until the percentage of waste plastic substitution of 40%, beyond which workability declined. Saikia et al. [52] found that the replacement of 5%, 10%, and 15% in volume of the natural aggregate produced a reduction in the workability of 6% when the plastic aggregates have an angular shape and an increase of 4% for the replacement of 15%. In [119], the workability of concrete increased by about 40% with plastic powder and it decreased by about 60% with plastic fibers.

Kassa et al. [120] evaluated the workability of concretes reinforced with PET fibers 100 mm long and 2 mm width with AR equal to a value of 50 and discovered that PET fibers affect the workability negatively because the latter drastically decreased when the PET fibers have been added in a percentage of 0.5%, 1.0%, and 1.5%. The value of the workability of concrete also decreased [121] when using PET reinforcement as aggregate due to the lack of hydration as well as segregation (caused by poor adhesion between the binder and the agglomerate [52]).

The hydrophobicity of PET reduces bonding with the cement slurry, generating voids and reducing workability [122]. As the PET content increased, the fresh concrete plasticity and consistency decreased. The decreasing fall is also attributed to PET aggregates with sharper edges than natural aggregates [123] because angular PET shapes increase the friction between particles leading to less workability in the mixtures [124]. On the contrary, according to [56], the smooth surface and hydrophobic nature (almost zero water absorption capacity) of plastics led to an increased slump value due to the reduced internal friction between the plastic particles and the cement matrix. Regarding the shape of PET reinforcement, fibers are usually more detrimental to workability than particles [125,126].

Air content is an important parameter since the high porosity causes an increase in water absorption which has a detrimental effect on concrete and mortar [127]. Generally, the implementation of plastic aggregates such as PET within cement mixtures produces a slight increase in air content as the replacement ratio increases [128]. The higher air content is attributed to the tendency of plastic particles to entrap more air in the microcracks and between the folds of the pieces [129]. In addition, plastic has a lower density and flat surfaces with sharp edges which allowed more air to be entrapped [130].

### 5.3. Influence on the Hardened Properties

The use of PET material in concrete and mortar produces a reduction in weight and, as a result, in density [131], because plastic materials typically weigh less than organic aggregates (e.g., sand) [132]. Hence, the relationship between the unit weight of plastic-modified mixtures and plastic aggregate content is linear with a decreasing trend [5,133].

The replacement of a certain volume of aggregates with an equivalent volume of PET, as fibers or aggregates, helps to reduce the overall density of the mix [134]. In [135], the addition of PET fibers led to a slight decrease in density; this reduction remains below 2.0%, even with a fiber content of 3.0%.

Moreover, the temperature can affect the density; in fact, as shown in the literature [50,100,136], the increase in temperature significantly decreases this property. The main advantage of the density reduction is that lightweight concrete in a structure contributes to a decrease its inertial force and, as a result, the earthquake risk of a building [33].

PET is a waterproof material, for this reason it has a low water absorption capacity [59,107]. Moreover, PET is also a material that neither mixes with the aggregates nor binds with the water in the cement mixtures, so it remains segregated and reduces the workability of the final conglomerate. The weak bonding between PET and cement [59], due to the hydrophobicity of the plastic, is also the cause of an uneven and inconsistent mixture [137]. For this reason, the workability, compactness, and sorptivity of the cement mixes with PET decrease [100]. The reduction in sorptivity is an advantageous aspect for the durability of reinforced concrete structures [138]. Molten PET can form an impermeable layer around natural aggregates ensuring less water absorption. In this way, even though the natural aggregates produce greater porosity, the degree of water absorption of the PET-reinforced mix is much less than that without PET [139]. On the contrary, other researchers [33,55,140] have stated that when using waste PET aggregates with a flaky shape or fine-grain form, along with the air content, water absorption also increases. The high-water absorption, a consequence of the gain in the porosity of the mortar, enables water to infiltrate into the cementitious mixture more easily.

Drying shrinkage of concretes and mortars is caused by the evaporation of the water from the mixtures. This mechanism is one of the main causes of cracking and the increase in the permeability of cement-based materials [141]. For this reason, it is important to understand whether the addition of PET reinforcement can reduce this criticality. Several studies [142,143,144] show that waste PET reinforcement can reduce drying shrinkage, increasing the compactness of the cementitious conglomerate. In fact, polyethylene terephthalate recycled fibers from PET waste bottles can be used to control shrinkage cracks in concrete and mortar [145,146,147]. Fibers with a higher aspect ratio are generally more effective at controlling cracking [148].

A significant improvement in minimizing the plastic shrinkage cracking of mortar was observed in [95] by increasing the fiber volume fraction over a range from 1.0% to 1.50% for a PET fiber length of 50 mm. The plastic shrinkage cracks disappeared completely at a fiber volume fraction equal to 1.5% and fiber length of 50 mm.

The advantage of reducing conglomerate shrinkage is the significant increase in the durability of the material [144,146].

A lower cracking allows a higher concrete part to be considered in the absorption of loads. The effective reagent part of the concrete increases. Within a reinforced concrete section, it is possible to consider non-cracked stiffness, which improves the tensile response of the loaded section. Finally, the smaller opening of the concrete cover cracks produces a better protection of the reinforcing steel against the ingress of harmful substances such as chlorides or CO_2_.

The compressive strength values of these new reinforced materials with PET can be considered the main critical aspect because the organic aggregate generally has a higher compressive strength of waste plastic. Thus, partially, or totally replacing a stronger aggregate (sand) with a less strong aggregate (PET) results in an overall less compressive strength for the cement mixture [5,149,150,151]. However, using PET particles with small dimensions [152,153,154] and fairly regular shapes, or longer, deformed PET fibers [82], a better compressive strength comparable to that of concrete without PET can be reached [50,99].

**Figure 8:**
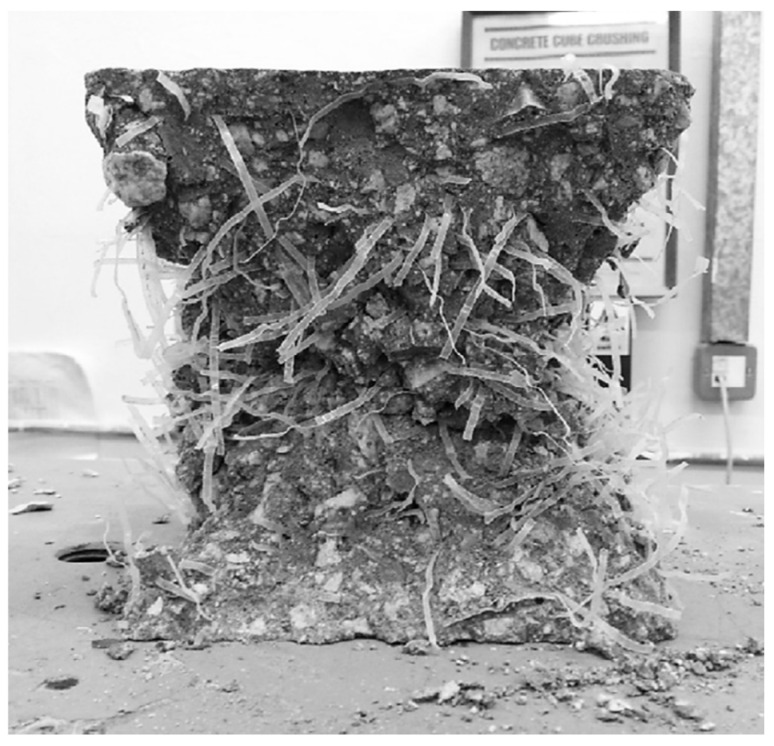
Deformed fiber-reinforced cube after failure [82].

**Figure 9:**
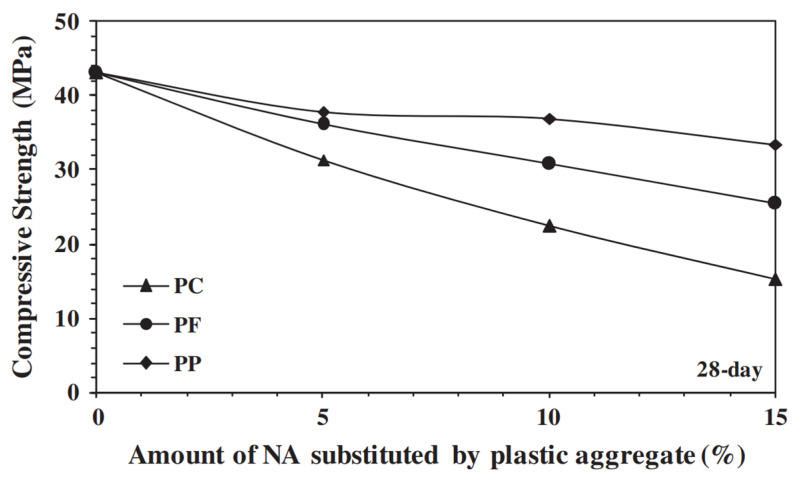
Compressive strength of concrete versus incorporation of PET aggregate to replace natural aggregate [52].

Figure 8 shows an image of a cubic sample of concrete reinforced with deformable PET fibers broken by compression. In Figure 9, meanwhile, it is possible to see the reduction in the 28-day compressive strength of concrete when the amount of PET increases as its geometry changes.

In relation to the curing environment, Ferreira [50] showed how the laboratory conditions or the wet chamber curing guarantee higher compressive strength values compared to outdoor environment curing. Anyway, as plastic waste aggregates are incorporated into the concrete, its compressive strength, splitting tensile strength, and modulus of elasticity decrease, regardless of the type and curing time, or the plastic type.

The weight substitution limit of PET fibers for which there can be a slight increase in the compressive strength of the material corresponds to 5%; after this threshold, there is only a gradual reduction in strength [51]. In [115], for 1% of 10 mm PET fibers, the cubic compressive strength shows a little increase up to 4%vol. of the original strength, while up to 3%vol., the cubic compressive strength shows a 20% reduction.

In summary, compressive strength slightly decreases with the increase in the waste material and in the AR. This reduction depends on the weakness of the internal structure of the concrete, which causes large voids around the fibers [91].

Some studies state that the use of fine and coarse PET aggregates in a cementitious mixture produces an increasing reduction in flexural strength as the percentage of plastic replacement increases [154]. The reduction in flexural strength is attributed to a decrease in adhesive strength between the surface of waste plastic and the cement matrix [155].

Moreover, Dawood’s studies [156] show that increasing the weight in percentage of fine and coarse PET particles from 5% to 10% increases the flexural strength to 27.15% and 30.24%, respectively. Similar results were also confirmed in [45,46,127]. The flexural strength decreases when the replacement ratio exceeds 10%. This reduction is caused by the fact that PET particles, being less weighty than natural aggregates, become concentrated in certain regions of the specimen by accumulating in specific groups. These groups represent weak points for the conglomerate and, therefore, the areas where specimen failure begins [156,157].

Moreover, the use of PET fibers, that have a higher tensile strength than natural aggregates, can increase the tensile strength (and thus the flexural strength) of cementitious mixtures. This increase can be explained by the fact that plastic fibers work to increase the bonding of concrete components and operate with a principle similar to the reinforcing process acting as a conveyor medium for stresses in the cracking area [91]. In [158], the fibers (30 mm long and 3 mm wide), produced from recycled PET materials and introduced into concrete at a volume percentage of 1%, increased flexural strength by 9.50%. As demonstrated by [159], flexural strength is proportional to the increase in the PET fiber content of the concrete mix. These fibers produce a behavior similar to that produced by steel fibers in the mix; in fact, they delay the drying shrinkage and improve the connection of the cementitious paste [160].

The temperature can positively affect the flexural strength of PET fiber-reinforced mortars because heating them to a temperature close to the melting temperature of PET (260 °C) results in much higher flexural strengths. However, at 400°, these strengths drop sharply because of the voids that are generated after the PET has melted, which limit the adhesion between the fiber and matrix, causing poor tensile stress transfer [100,161]. The use of fine PET as aggregates (max size of 4 mm) combined with the increase in the temperature from 100 °C to 400 °C leads to a reduction in the flexural and compressive strength ranging from 30% to 40% [161].

The high value of the tensile strength of PET contributes to increase the ultimate strain at break of the cementitious mixture [162] and reduce the cracking phenomena [163].

**Figure 10:**
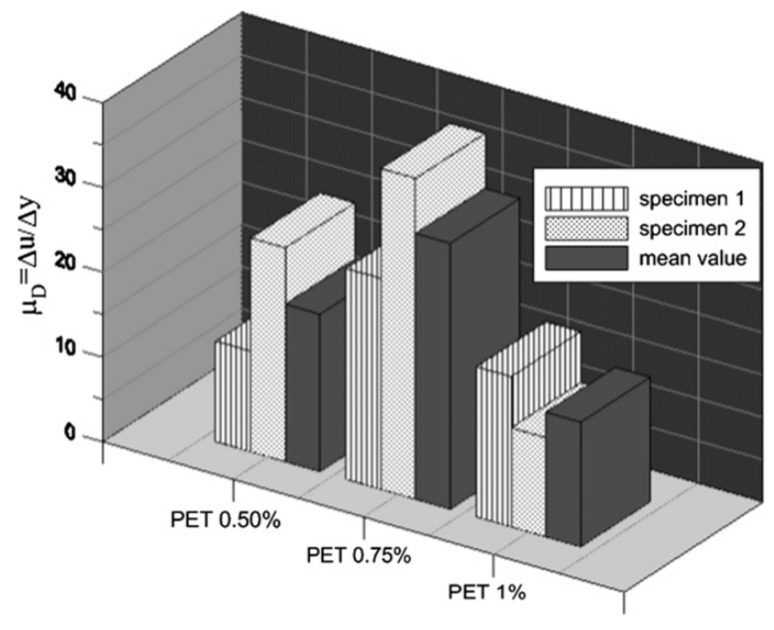
Values of μ_d_ (=ductility) for the three different fiber contents. Laminated fibrous reinforcement [164].

The first effect of the increase in ultimate strain at failure is the rise in ductility of the cement mixture. As shown in Figure 10, Foti [164] demonstrated that the ductility value changes significantly as the percentage in weight of fiber varies. In particular, using circular PET fibers with 0.75% in weight, the ductility of concrete reached the value of 37.88, while with 1%, it reached the value of 11.73.

The ductility equation is:

(3) $$\mu_{d}=\frac{\Delta u}{\Delta y}$$

where Δu is the maximum deformation at the centerline and Δy is the deflection at the peak load.

**Figure 11:**
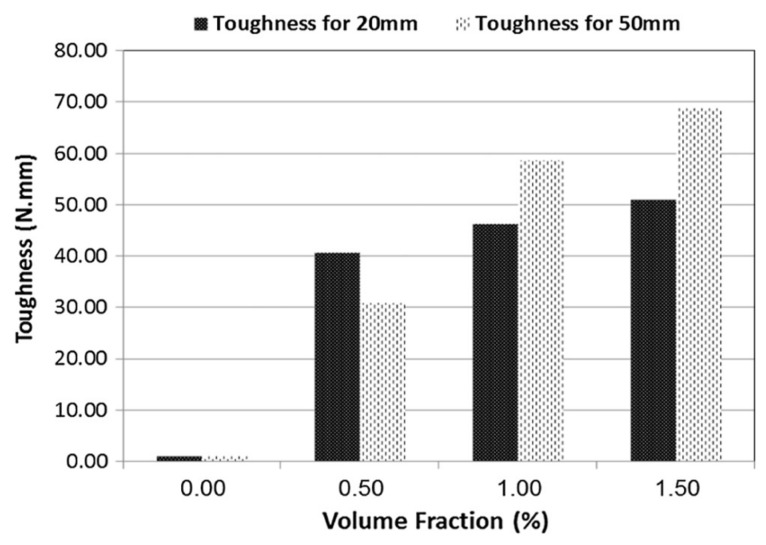
Comparison of toughness for mortar mixtures with increasing percentage of volume fraction of PET fibers [95].

The second effect of the increase in ultimate strain at failure, so, in ductility, is the gain of the toughness [165]. In [95], due to the fibers bridging cracks in the matrix, the toughness of fiber-reinforced mortar was significantly higher than that of plain mortar. The fiber volume fraction positively affects toughness [166,167]. In [95], increasing the fiber volume fraction up to 1.5% resulted in a substantial increase in toughness. In Figure 11, it is shown that fibers 20 mm long at 0.5%, 1.0%, and 1.5% volume fractions produce increases in toughness of 3.570%, 4.056%, and 4.492%, respectively, compared to fiber-free mortar, where the increases in toughness, for fibers 50 mm long, were 2.685%, 5.192%, and 6.107%, respectively [95].

Asdollah-Tabar et al. [168] calculated the fracture toughness (*K_Ic_*) using the following relationship:

(4) $$K_{Ic}=\frac{P_{c}\sqrt{a}}{RB\sqrt{\pi }}Y_{I}(a,R)$$

where $P_{c}$ is the maximum failure load, R is the disk radius, a is the half crack length, B is the sample thickness, and $Y_{I}$ is the geometry factor that is a function of crack length to radius ratio (a/R).

In [168], the fracture toughness increased up to 8.5% and 16.3% when a percentage of 4% in weight of fine and coarse aggregates were added, respectively. The impact of the addition of coarse aggregates was more significant than the fine one due to the resistance produced by the coarse PET aggregate. In general, PET aggregates produce resistance in front of crack propagation and do not allow the crack to grow readily, and this consequently causes an increase in the fracture toughness. In general, it can be stated that the increased plastic energy of PET plastic aggregates leads to enhanced energy absorption properties in the concrete [169].

**Figure 12:**
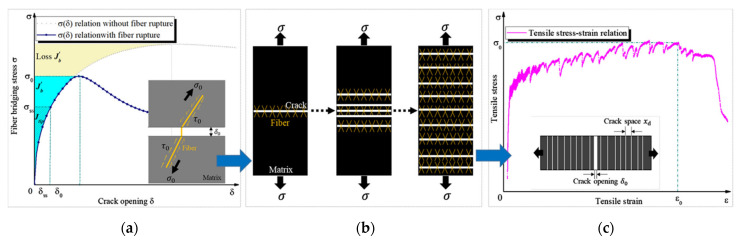
Schematic diagram of the bridging stress for multiple cracking and the resulting high ductility behavior. (**a**) Bridging stress—single crack opening; (**b**) fiber bridging stress transfer; and (**c**) composite tensile stress–strain [172].

The process that leads to an increase in overall ductility (and slight increase in tensile strength) of the PET fiber-reinforced cementitious mixture is described in Figure 12. When the cement matrix is stressed by tensile stresses, it cracks. This crack does not continue to open but sews its fracture as all the tensile stress $\sigma_{0}$ is absorbed by the bonding fiber.

Ensuring that there is a perfect adhesion between the fiber and matrix, $\sigma_{0}$, the tensile stress, must be balanced and transferred to the matrix itself through the $\tau_{0}$ contact forces generated along the outer surface of the fiber. Thus, for the balance of the internal forces in the material, the $\tau_{0}$ shear stress developed in the fiber surroundings will have to be balanced by other stresses. These stresses are those that arise in the cement matrix and produce other cracks in the mixture. Progressive cracks will be generated (Figure 12b) [170] and their opening will be contained by other fibers, ensuring an overall greater ultimate strain (Figure 12c). To ensure the transfer of the tensile stress within multiple sections of the cement matrix, two requirements must be satisfied [171]:

(I) The strength criterion, which is expressed by the relationship $\sigma_{0}\geq \sigma_{cr}$ according to which the contact surface between fiber and matrix must be strong enough to ensure the transfer of stresses from one material to the other. In (5), $\sigma_{0}$ is the tensile strength of the fiber and $\sigma_{cr}$ is the tensile cracking strength of the cement matrix.

(II) The energy criterion, which is expressed by the relationship:

(5) $${J\prime }_{b}=\sigma_{0}\delta_{0}-\int_{0}^{\delta_{0}}\sigma \left(\delta \right)d\delta \geq J_{tip}$$

according to which the crack tip toughness $J_{tip}$ must be less than the energy ${J\prime }_{b}$, which corresponds to the development of more cracks.

## 6. Discussion and Conclusions

### 6.1. Remarks

Although scientific research has explored various aspects concerning the recycling of plastic materials like PET as reinforcement of cementitious mixtures, there are still several unexplored areas of this topic.

- The study of the interaction between PET and cement in order to compact the reinforcement with the matrix, minimizing the number of internal voids. No researcher has yet found an additive that chemically bonds cement with plastic. Moreover, the hydrophobic nature of PET makes it not so compatible with mixtures that harden in the presence of water. Therefore, an additive should be found that reduces the hydrophobicity of PET, enhancing the chemical interaction between it and the cement matrix.
- Experimentally, there are several scientific studies performed on PET-reinforced specimens where their performances were compared with equivalent specimens without PET. However, there is a lack of real examples of existing constructions made with these cementitious mixtures reinforced with recycled PET. By building simple concrete structures reinforced with PET, it would be possible to appreciate its effectiveness in the real operating conditions of a building.
- The industrialization of the cutting of PET into aggregates or fibers with a precise, constant geometry is a goal that construction companies have not yet achieved. The capability of designing the aspect ratio of a fiber or the equivalent diameter of a plastic aggregate would allow the serial production of the reinforcement material. Machines would cut a product that would always be the same. Therefore, the geometric properties of the reinforcement would remain constant and known to the engineer. With this background, it would be possible to avoid all mistakes related to the imperfections resulting from the manual cutting of the recycled product.
- Research in this area investigates the reuse of PET as a reinforcing material applied in the cement mixture only during the mixing phase but never in the production phase of the individual components. Plastic waste could be introduced in the cement production phase to build a sustainable binder. In the same way, natural aggregate could be processed through a thin layer of liquefied PET. By bringing the plastic to melting temperature, it would be easy to cast on common concrete aggregates. These new binders or aggregates would then be mixed with plastic during the earliest stages of production of the individual material that makes up the cement mix. Cementitious specimens made from these new mix designs could be tested to evaluate their properties in the fresh and hardened states.
- The latest scientific findings reveal that plastic has gradually contaminated every ecosystem. Microplastics are everywhere. Over time, the cyclic loads that PET plastic waste underwent in marine environments caused the rocks of the seabed to bind to the waste. The formation of these new materials known as plastiglomerates, pyroplastics, and plasticrusts may become a useful resource for the creation of new sustainable building materials. Climatic chambers could be used to reproduce the thermal and pressure stresses that PET underwent as it sedimented on the seabed. Within a short time, it would be possible to create these new materials that blend waste plastic with natural rocks or recycled aggregates from construction waste.

Several studies have shown how PET in an alkaline environment can deteriorate by losing part of its original characteristics. Chemical investigations of molecular structures can predict the long-term behavior of PET in an alkaline environment. However, only experimental investigations carried out on cement-reinforced PET specimens can confirm whether this material undergoes a decay in mechanical performance in the long-term. For future studies, it is recommended to use accelerated ageing systems that simulate the ageing process of the specimens by subjecting them to high temperatures, freeze/thaw cycles, and chemical exposure. Comparing the performance of aged specimens with virgin ones will make it possible to evaluate the performance of PET reinforcement within cementitious mixtures over time.

### 6.2. Conclusions

The use of PET offers several changes in the physical and mechanical properties of cementitious mixtures. Depending on the specific use of mortars or concretes reinforced with waste plastic, different dimensional geometries and amounts of PET reinforcement may be adopted to improve certain properties rather than others.

This article discusses the most recent studies on the reinforcement of cementitious mixtures with PET introduced in different forms, with aggregate or fiber function. It was seen how each property in the fresh state or in the hardened state changes in relation to the size and specific function that PET takes on in the mixture.

This review focused on the most important fresh-state physical properties of a mortar or concrete mix reinforced with waste PET materials, i.e., workability and air content. Then, the properties in the hardened state (density, water absorption, and shrinkage) that affect the mechanical performance (compressive strength, flexural strength, ductility, and toughness) of the materials were analyzed. In addition, other equally significant characteristics of cementitious mixtures could be investigated, such as carbonatation, abrasion resistance, and hardness.

For the same PET waste plastic material, under the same temperature and pressure conditions, it was observed that conglomerate characteristics changed with respect to the following geometric quantities of a single PET element: (I) length l; (II) width w; (III) thickness t; (IV) equivalent diameter $d_{e}$; and (V) quantity $\frac{\%}{100}$.

The interaction existing between the PET plastic aggregate and the cement matrix also depends on the shape of the aggregate and the existing relationship between its dimensions. This happens both when PET is an aggregate and when it is a fiber. However, when PET is an aggregate, the two shapes most used in studies are rougher and angular and spherical and smoother. When PET is a fiber, on the other hand, the parameter that influences the interaction between the materials is the aspect ratio (AR).

It is possible to compare the reinforcing function of PET to the reinforcing function of steel in concrete [173], although the two materials do not have the same ability to interact together. In fact, while steel has a good chemical affinity with concrete, PET has dissimilarities. It can be stated that the reduction in mechanical properties of PET fiber-reinforced cement composites depends on the poor bonding between the cement and waste plastic [59,122,174] and on the weak affinity between plastic and water, which is repelled from the cementitious matrix [51]. However, although most experimental evidence does not demonstrate chemical affinities between PET and the cementitious matrix, the effectiveness of plastic reinforcement results from the shape–mechanical interaction it exhibits with cementitious mixtures. For this reason, it is possible to summarize the benefits of PET reinforcement in mortars and concretes as follows:

- Workability increases with smooth, circular PET aggregates because of the reduction in friction existing between the plastic and cementitious matrix.
- As the percentage of PET increases, the air content tends to increase since plastic has a hydrophobic nature, so it does not bind with water, leaving porosity in the mix.
- Density tends to decrease as the percentage of PET increases since it is a lighter material than natural aggregate. Weight reduction is a great advantage for the structural material as it saves costs and provides structural lightness. The latter aspect underlies the reduction in inertial force and is therefore critical in seismic zones, where seismic action is directly proportional to the increase in structural mass.
- Water absorption is generally reduced due to the hydrophobic nature of PET. However, by using flaky particles, it is possible to have an increase in water absorption due to the increase in porosity, which produces an increase in the volume of voids within which water can easily infiltrate.
- The contribution of PET in the form of fibers produces a reduction in material shrinkage because the interaction between the plastic and cement matrix can hold back the opening of any cracks produced by shrinkage. In fact, a PET fiber acts as a seam when two edges of the mix move apart. This is a big advantage in that it produces an increase in the durability of mortars and concretes.
- By using PET in the form of particles with small sizes and a regular shapes, a slight increase in compressive strength can be achieved. Generally, the threshold limit value for obtaining benefits in terms of mechanical strength is a percentage equal to 5% by weight.
- While the introduction in the form of PET aggregate does not always produce an increase in flexural strength, the use of fibers always succeeds in increasing this parameter. The behavior that these fibers produce is like that produced by steel rods within reinforced concrete. In addition, the increase in temperature below the melting temperature of PET has a beneficial effect in increasing the flexural strength.
- The stitching effect of PET fibers is able to restrain the opening of cracks when the concrete mix goes into tension, delaying failure. This property increases both ductility and toughness.
- Comparing the CO_2_ emissions associated with the production and transport of natural aggregates used to manufacture concrete mixes with those of PET plastic aggregates, it can be stated that this solution is more environmentally sustainable. It was found that the CO_2_ consumption of a conventional concrete building is higher than that of the corresponding concrete building with PET.

Finally, the reinforcement by PET, produced from plastic waste materials (such as plastic bottles), is a useful strategy for improving the characteristics of mortars and concretes. The rational introduction of PET elements in the form of fibers or aggregates designed with a specific geometry could be a sustainable solution to reduce the ever-increasing amount of plastic in landfills. Furthermore, its employment is an interesting alternative to using a material with reduced durability and high specific weight such as steel.

## Figures and Tables

**Table 1:** Mechanical properties of virgin and recycled PET fibers [25].

Property	Virgin PET Fiber	Recycled PET Fiber
Density ^1^ (kg/m^3^)	1390	1356
Elastic modulus (MPa)	5690	10,500
Tensile strength (MPa)	140.5	220.0
Breaking stress (kg/cm^2^)	82.2	42.2
Break elongation (%)	6.96	5.00

^1^ Density values of virgin and recycled PET obtained from [26] and [25,27,28,29], respectively.

**Table 2:** Proportions of concrete mix design and CO_2_ emissions [44].

Raw Materials	Weight per m^3^ of Concrete (kg/m^3^)	CO_2_ Emissions (kgCO_2_/kg concrete)
Portland cement type II	495	0.885
Coarse aggregates	899.3	0.0075
Fine aggregates	819.8	0.0026
Water	165	0.000196
PET	Depends on the replacement ratio	−3.38

**Table 3:** Total CO_2_ consumption for each mixture.

		CO_2_ Emissions (kgCO_2_/m^3^)	Global CO_2_ Emissions (tCO_2_)	Concrete CO_2_ Emissions (tCO_2_)
Scenario 0	Portland cement type II	438.08	80.459	82.095
	Coarse aggregates	6.74	1.239	
	Fine aggregates	2.13	0.391	
	Water	0.03	0.006	
	0% PET	0.00	0.000	
Scenario 1	Portland cement type II	416.17	76.436	62.708
	Coarse aggregates	6.74	1.239	
	Fine aggregates	2.13	0.391	
	Water	0.03	0.006	
	5% PET of cement	−83.66	−15.364	
Scenario 2	Portland cement type II	438.08	80.459	56.629
	Coarse aggregates	6.74	1.239	
	Fine aggregates	2.02	0.372	
	Water	0.03	0.006	
	5% PET of aggregates	−138.55	−25.446	
Scenario 3	Portland cement type II	427.12	78.447	59.669
	Coarse aggregates	6.74	1.239	
	Fine aggregates	2.08	0.382	
	Water	0.03	0.006	
	5% PET of cement + 2.5% PET of aggregates	−111.10	−20.405	

## References

[1] UNEP (2018). The State of Plastics World Environment Day Outlook.

[2] Singh N., Hui D., Singh R., Ahuja I.P.S., Feo L., Fraternali F. (2017). Recycling of Plastic Solid Waste: A State of Art Review and Future Applications. Compos. B Eng..

[3] Da Cruz N.F., Ferreira S., Cabral M., Simões P., Marques R.C. (2014). Packaging Waste Recycling in Europe: Is the Industry Paying for It?. Waste Manag..

[4] Arulrajah A., Perera S., Wong Y.C., Horpibulsuk S., Maghool F. (2020). Stiffness and Flexural Strength Evaluation of Cement Stabilized PET Blends with Demolition Wastes. Constr. Build. Mater..

[5] Coviello C.G., Lassandro P., Sabbà M.F., Foti D. (2023). Mechanical and Thermal Effects of Using Fine Recycled PET Aggregates in Common Screeds. Sustainability.

[6] Ahdal A.Q., Amrani M.A., Ghaleb A.A.A., Abadel A.A., Alghamdi H., Alamri M., Wasim M., Shameeri M. (2022). Mechanical Performance and Feasibility Analysis of Green Concrete Prepared with Local Natural Zeolite and Waste PET Plastic Fibers as Cement Replacements. Case Stud. Constr. Mater..

[7] Vedrtnam A., Bedon C., Barluenga G. (2020). Study on the Compressive Behaviour of Sustainable Cement-Based Composites under One-Hour of Direct Flame Exposure. Sustainability.

[8] Bamigboye G.O., Olukanni D.O., Adedeji A.A., Ojewumi M.O., Jolayemi K.J. (2018). Experimental and Modelling of Flexural Strength Produced from Granite-Gravel Combination in Self-Compacting Concrete. Int. J. Civ. Eng. Technol..

[9] Hussain J., Khan A., Zhou K. (2020). The Impact of Natural Resource Depletion on Energy Use and CO_2_ Emission in Belt & Road Initiative Countries: A Cross-Country Analysis. Energy.

[10] Mohamad N., Muthusamy K., Embong R., Kusbiantoro A., Hashim M.H. (2022). Environmental Impact of Cement Production and Solutions: A Review. Mater. Today Proc..

[11] Wi K., Lee H.-S., Lim S., Song H., Hussin M.W., Ismail M.A. (2018). Use of an Agricultural By-Product, Nano Sized Palm Oil Fuel Ash as a Supplementary Cementitious Material. Constr. Build. Mater..

[12] Blankendaal T., Schuur P., Voordijk H. (2014). Reducing the Environmental Impact of Concrete and Asphalt: A Scenario Approach. J. Clean. Prod..

[13] Shaikh F.U.A. (2020). Tensile and Flexural Behaviour of Recycled Polyethylene Terephthalate (PET) Fibre Reinforced Geopolymer Composites. Constr. Build. Mater..

[14] Coviello C.G., Sabbà M.F., Foti D. (2024). Recycled Waste PET for Sustainable Cementitious Materials. Reference Module in Materials Science and Materials Engineering.

[15] Mendivil-Escalante J.M., Gómez-Soberón J.M., Almaral-Sánchez J.L., Cabrera-Covarrubias F.G. (2017). Metamorphosis in the Porosity of Recycled Concretes through the Use of a Recycled Polyethylene Terephthalate (PET) Additive. Correlations between the Porous Network and Concrete Properties. Materials.

[16] Meawad A., Ibrahim S. (2019). Novel Bifunctional Dispersing Agents from Waste PET Packaging Materials and Interaction with Cement. Waste Manag..

[17] Si J., Hu J., Liu Z., Zeng Z., Li K., Ding X. (2023). Experimental Study on Flexural Performance of Reinforced Concrete Beams Strengthened by PET. Struct. Concr..

[18] Rasheed L.S., Shaban A.M., Abdulrasool A.T. (2022). Mechanical and Structural Characteristics of PET Fiber Reinforced Concrete Plates. Smart Sci..

[19] Kumar M.L.A., Anoop R., Ramana I.V., Sasidhar C. (2014). Experimental Investigations on the Flexural Strength of PET Reinforced Concrete. Int. J. Emerg. Technol. Adv. Eng..

[20] Al-Gabri B.N.A., Nabilah A.B., Abdul Aziz F.N.A., Karim I.A. (2019). Numerical Analysis of Out-of-Plane Deformation of Shear Wall. Proc. IOP Conf. Ser. Earth Environ. Sci..

[21] Skibicki S., Pułtorak M., Kaszyńska M., Hoffmann M., Ekiert E., Sibera D. (2022). The Effect of Using Recycled PET Aggregates on Mechanical and Durability Properties of 3D Printed Mortar. Constr. Build. Mater..

[22] Szpetulski J., Stawiski B., Witkowski P. (2022). Tests Regarding the Effect of Dispersed Reinforcement Made with a Prototype Device from PET Beverage Bottles on the Strength Properties of Concrete. Energy.

[23] Coviello C.G., Mansour S., Rizzo F., Foti D. (2023). An Experimental Study on the Mechanical and Thermal Properties of Graphene-Reinforced Screeds. Proceedings of the Fib Symposium.

[24] Sayı C.Ö., Eren Ö. (2022). Physical and Durability Properties of Recycled Polyethylene Terephthalate (PET) Fibre Reinforced Concrete. Eur. J. Environ. Civ. Eng..

[25] Tapia-Picazo J.C., Luna-Bárcenas J.G., García-Chávez A., Gonzalez-Nuñez R., Bonilla-Petriciolet A., Alvarez-Castillo A. (2014). Polyester Fiber Production Using Virgin and Recycled PET. Fibers Polym..

[26] Avinc O., Khoddami A. (2009). Overview of Poly(Lactic Acid) (PLA) Fibre. Fibre Chem..

[27] Usman N., Masirin M.I.M., Ahmad K.A., Ali A.S.B. (2019). Application of Recycled Polyethylene Terephthalate Fiber in Asphaltic Mix for Fatigue Life Improvement. Lecture Notes in Civil Engineering.

[28] Lambert S., Wagner M. (2018). Microplastics Are Contaminants of Emerging Concern in Freshwater Environments: An Overview. Handbook of Environmental Chemistry.

[29] Lusher A., Hollman P., Mendoza-Hill J. (2017). Microplastics in Fisheries and Aquaculture: Status of Knowledge on Their Occurrence and Implications for Aquatic Organisms and Food Safety.

[30] Askar M.K., Al-Kamaki Y.S.S., Hassan A. (2023). Utilizing Polyethylene Terephthalate PET in Concrete: A Review. Polymers.

[31] Rahimi S.R., Nikbin I.M., Allahyari H., Habibi T.S. (2016). Sustainable Approach for Recycling Waste Tire Rubber and Polyethylene Terephthalate (PET) to Produce Green Concrete with Resistance against Sulfuric Acid Attack. J. Clean. Prod..

[32] Tang R., Wei Q., Zhang K., Jiang S., Shen Z., Zhang Y., Chow C.W.K. (2022). Preparation and Performance Analysis of Recycled PET Fiber Reinforced Recycled Foamed Concrete. J. Build. Eng..

[33] Akçaözoǧlu S., Atiş C.D., Akçaözoǧlu K. (2010). An Investigation on the Use of Shredded Waste PET Bottles as Aggregate in Lightweight Concrete. Waste Manag..

[34] Pereira De Oliveira L.A., Castro-Gomes J.P. (2011). Physical and Mechanical Behaviour of Recycled PET Fibre Reinforced Mortar. Constr. Build. Mater..

[35] Shen W., Cao L., Li Q., Zhang W., Wang G., Li C. (2015). Quantifying CO_2_ Emissions from China’s Cement Industry. Renew. Sustain. Energy Rev..

[36] Bostanci S.C., Limbachiya M., Kew H. (2018). Use of Recycled Aggregates for Low Carbon and Cost Effective Concrete Construction. J. Clean. Prod..

[37] Chaudhury R., Sharma U., Thapliyal P.C., Singh L.P. (2023). Low-CO_2_ Emission Strategies to Achieve Net Zero Target in Cement Sector. J. Clean. Prod..

[38] Huesca-Tortosa J.A., Spairani-Berrio Y., Coviello C.G., Sabbà M.F., Rizzo F., Foti D. (2024). Evaluation of Eco-Friendly Consolidating Treatments in Pugliese Tuff (Gravina Calcarenite) Used in Italian Heritage Buildings. Buildings.

[39] Fayomi G.U., Mini S.E., Fayomi O.S.I., Ayoola A.A. (2019). Perspectives on Environmental CO_2_ Emission and Energy Factor in Cement Industry. Proc. IOP Conf. Ser. Earth Environ. Sci..

[40] Nie S., Zhou J., Yang F., Lan M., Li J., Zhang Z., Chen Z., Xu M., Li H., Sanjayan J.G. (2022). Analysis of Theoretical Carbon Dioxide Emissions from Cement Production: Methodology and Application. J. Clean. Prod..

[41] Meddah M.S. (2017). Recycled Aggregates in Concrete Production: Engineering Properties and Environmental Impact. Proceedings of the MATEC Web of Conferences.

[42] Andrew R.M. (2018). Global CO_2_ Emissions from Cement Production. Earth Syst. Sci. Data.

[43] Usahanunth N., Pochanart P. (2017). CO_2_-Emission Assessment of the Concrete Added Crushed PET Bottles Waste. Interdiscip. Res. Rev..

[44] Nikbin I.M., Dezhampanah S., Charkhtab S., Mehdipour S., Shahvareh I., Ebrahimi M., Pournasir A., Pourghorban H. (2022). Life Cycle Assessment and Mechanical Properties of High Strength Steel Fiber Reinforced Concrete Containing Waste PET Bottle. Constr. Build. Mater..

[45] Rahmani E., Dehestani M., Beygi M.H.A., Allahyari H., Nikbin I.M. (2013). On the Mechanical Properties of Concrete Containing Waste PET Particles. Constr. Build. Mater..

[46] Asadi S.S. (2017). Pet Bottle Waste as a Supplement to Concrete Fine Aggregate. Int. J. Civ. Eng. Technol..

[47] Huang S., Wang H., Ahmad W., Ahmad A., Ivanovich Vatin N., Mohamed A.M., Deifalla A.F., Mehmood I. (2022). Plastic Waste Management Strategies and Their Environmental Aspects: A Scientometric Analysis and Comprehensive Review. Int. J. Environ. Res. Public Health.

[48] da Silva T.R., de Azevedo A.R.G., Cecchin D., Marvila M.T., Amran M., Fediuk R., Vatin N., Karelina M., Klyuev S., Szelag M. (2021). Application of Plastic Wastes in Construction Materials: A Review Using the Concept of Life-Cycle Assessment in the Context of Recent Research for Future Perspectives. Materials.

[49] Caballero-Jorna M., Serna P., Roig-Flores M., Mechtcherine V., Signorini C., Junger D. (2024). Determining the Effects of Extreme Environmental Conditions on the Ageing of Macro Synthetic Fiber Reinforced Concrete: A Statistical and Analytical Study. Proceedings of the Transforming Construction: Advances in Fiber Reinforced Concrete.

[50] Ferreira L., De Brito J., Saikia N. (2012). Influence of Curing Conditions on the Mechanical Performance of Concrete Containing Recycled Plastic Aggregate. Constr. Build. Mater..

[51] Abed J.M., Khaleel B.A., Aldabagh I.S., Sor N.H. (2021). The Effect of Recycled Plastic Waste Polyethylene Terephthalate (PET) on Characteristics of Cement Mortar. Proc. J. Phys. Conf. Ser..

[52] Saikia N., De Brito J. (2014). Mechanical Properties and Abrasion Behaviour of Concrete Containing Shredded PET Bottle Waste as a Partial Substitution of Natural Aggregate. Constr. Build. Mater..

[53] Fakhruddin, Irmawaty R., Djamaluddin R. (2022). Flexural Behavior of Monolith and Hybrid Concrete Beams Produced through the Partial Replacement of Coarse Aggregate with PET Waste. Structures.

[54] Choudhary R., Kumar A., Murkute K. (2018). Properties of Waste Polyethylene Terephthalate (PET) Modified Asphalt Mixes: Dependence on PET Size, PET Content, and Mixing Process. Period. Polytech. Civ. Eng..

[55] Kunthawatwong R., Sylisomchanh L., Pangdaeng S., Wongsa A., Sata V., Sukontasukkul P., Chindaprasirt P. (2022). Recycled Non-Biodegradable Polyethylene Terephthalate Waste as Fine Aggregate in Fly Ash Geopolymer and Cement Mortars. Constr. Build. Mater..

[56] Choi Y.W., Moon D.J., Chung J.S., Cho S.K. (2005). Effects of Waste PET Bottles Aggregate on the Properties of Concrete. Cem. Concr. Res..

[57] Sadrmomtazi A., Dolati-Milehsara S., Lotfi-Omran O., Sadeghi-Nik A. (2016). The Combined Effects of Waste Polyethylene Terephthalate (PET) Particles and Pozzolanic Materials on the Properties of Selfcompacting Concrete. J. Clean. Prod..

[58] Qaidi S., Al-Kamaki Y.S.S., Al-Mahaidi R., Mohammed A.S., Ahmed H.U., Zaid O., Althoey F., Ahmad J., Isleem H.F., Bennetts I. (2022). Investigation of the Effectiveness of CFRP Strengthening of Concrete Made with Recycled Waste PET Fine Plastic Aggregate. PLoS ONE.

[59] Albano C., Camacho N., Hernández M., Matheus A., Gutiérrez A. (2009). Influence of Content and Particle Size of Waste Pet Bottles on Concrete Behavior at Different w/c Ratios. Waste Manag..

[60] Hannawi K., Kamali-Bernard S., Prince W. (2010). Physical and Mechanical Properties of Mortars Containing PET and PC Waste Aggregates. Waste Manag..

[61] Da Silva A.M., De Brito J., Veiga R. (2014). Incorporation of Fine Plastic Aggregates in Rendering Mortars. Constr. Build. Mater..

[62] Janfeshan Araghi H., Nikbin I.M., Rahimi Reskati S., Rahmani E., Allahyari H. (2015). An Experimental Investigation on the Erosion Resistance of Concrete Containing Various PET Particles Percentages against Sulfuric Acid Attack. Constr. Build. Mater..

[63] Islam M.J., Meherier M.S., Islam A.K.M.R. (2016). Effects of Waste PET as Coarse Aggregate on the Fresh and Harden Properties of Concrete. Constr. Build. Mater..

[64] Perera S., Arulrajah A., Wong Y.C., Horpibulsuk S., Maghool F. (2019). Utilizing Recycled PET Blends with Demolition Wastes as Construction Materials. Constr. Build. Mater..

[65] Arulrajah A., Yaghoubi E., Wong Y.C., Horpibulsuk S. (2017). Recycled Plastic Granules and Demolition Wastes as Construction Materials: Resilient Moduli and Strength Characteristics. Constr. Build. Mater..

[66] Yaghoubi E., Arulrajah A., Wong Y.C., Horpibulsuk S. (2017). Stiffness Properties of Recycled Concrete Aggregate with Polyethylene Plastic Granules in Unbound Pavement Applications. J. Mater. Civ. Eng..

[67] Lee Z.H., Paul S.C., Kong S.Y., Susilawati S., Yang X. (2019). Modification of Waste Aggregate PET for Improving the Concrete Properties. Adv. Civ. Eng..

[68] Spósito F.A., Higuti R.T., Tashima M.M., Akasaki J.L., Melges J.L.P., Assunção C.C., Bortoletto M., Silva R.G., Fioriti C.F. (2020). Incorporation of PET Wastes in Rendering Mortars Based on Portland Cement/Hydrated Lime. J. Build. Eng..

[69] Foti D., Lerna M. (2020). New Mortar Mixes with Chemically Depolymerized Waste PET Aggregates. Adv. Mater. Sci. Eng..

[70] Khan M.I., Sutanto M.H., Khan K., Iqbal M., Napiah M.B., Zoorob S.E., Klemeš J.J., Bokhari A., Rafiq W. (2022). Effective Use of Recycled Waste PET in Cementitious Grouts for Developing Sustainable Semi-Flexible Pavement Surfacing Using Artificial Neural Network (ANN). J. Clean. Prod..

[71] Singh G., Kumar H., Singh S. (2019). Performance Evaluation-PET Resin Composite Composed of Red Mud, Fly Ash and Silica Fume. Constr. Build. Mater..

[72] Singh G., Kumar H., Singh S. (2020). Mechanical Performance of Red Mud Polymer Concrete Composites Fabricated Using Recycled PET Resin. Abstr. Int. Conf. Meet..

[73] Shirazimoghaddam S., Amin I., Faria Albanese J.A., Shiju N.R. (2023). Chemical Recycling of Used PET by Glycolysis Using Niobia-Based Catalysts. ACS Eng. Au.

[74] Waysal S.M., Patil Y.D., Dholkiya B.Z. (2022). Use of PET Resin and Metakaolin in Sustainable Production of Cement Mortar. Mater. Today Proc..

[75] Patil Y.D., Waysal S.M., Dholakiya B.Z. (2020). Feasibility of PET Resin as a Cement Substitute for Sustainable Construction. Proc. IOP Conf. Ser. Mater. Sci. Eng..

[76] Waysal S., Patil Y., Dholakiya B.K. (2023). Effect of PET Resin as Cement Substitute on Properties of Cement Mortar Subjected to Different During Conditions. U. Porto J. Eng..

[77] Hidaya N., Mutuku R.N., Mwero J.N. (2017). Physical and Mechanical Experimental Investigation of Concrete Incorporated with Polyethylene Terephthalate (PET) Fibers. Eur. Int. J. Sci. Technol..

[78] Lazorenko G., Kravchenko E., Kasprzhitskii A., Fini E.H. (2024). An Evaluation of the Environmental Impact and Energy Efficiency of Producing Geopolymer Mortar with Plastic Aggregates. Resour. Conserv. Recycl. Adv..

[79] Ayo A.W., Olukunle O.J., Adelabu D.J. (2017). Development of a Waste Plastic Shredding Machine. Int. J. Waste Resour..

[80] Rahim N.H.A., Khatib A.N.H.M. (2021). Development of PET Bottle Shredder Reverse Vending Machine. Int. J. Adv. Technol. Eng. Explor..

[81] Oyebade D., Okunola O., Olanrewaju O. (2019). Development of Shredding and Washing Machine for Polyethylene Terephthalate (PET) Bottles Pelletizer. Int. J. Eng. Sci. Appl..

[82] Borg R.P., Baldacchino O., Ferrara L. (2016). Early Age Performance and Mechanical Characteristics of Recycled PET Fibre Reinforced Concrete. Constr. Build. Mater..

[83] Kim S.B., Yi N.H., Kim H.Y., Kim J.H.J., Song Y.C. (2010). Material and Structural Performance Evaluation of Recycled PET Fiber Reinforced Concrete. Cem. Concr. Compos..

[84] Foti D. (2011). Preliminary Analysis of Concrete Reinforced with Waste Bottles PET Fibers. Constr. Build. Mater..

[85] Fraternali F., Spadea S., Berardi V.P. (2014). Effects of Recycled PET Fibres on the Mechanical Properties and Seawater Curing of Portland Cement-Based Concretes. Constr. Build. Mater..

[86] Corinaldesi V., Nardinocchi A. (2016). Influence of Type of Fibers on the Properties of High Performance Cement-Based Composites. Constr. Build. Mater..

[87] Fernández M.E., Payá J., Borrachero M.V., Soriano L., Mellado A., Monzó J. (2017). Degradation Process of Postconsumer Waste Bottle Fibers Used in Portland Cement–Based Composites. J. Mater. Civ. Eng..

[88] Poonyakan A., Rachakornkij M., Wecharatana M., Smittakorn W. (2018). Potential Use of Plastic Wastes for Low Thermal Conductivity Concrete. Materials.

[89] Shahidan S., Ranle N.A., Zuki S.S.M., Khalid F.S., Ridzuan A.R.M., Nazri F.M. (2018). Concrete Incorporated with Optimum Percentages of Recycled Polyethylene Terephthalate (PET) Bottle Fiber. Int. J. Integr. Eng..

[90] Alani A.H., Bunnori N.M., Noaman A.T., Majid T.A. (2019). Durability Performance of a Novel Ultra-High-Performance PET Green Concrete (UHPPGC). Constr. Build. Mater..

[91] Khatab H.R., Mohammed S.J., Hameed L.A. (2019). Mechanical Properties of Concrete Contain Waste Fibers of Plastic Straps. Proc. IOP Conf. Ser. Mater. Sci. Eng..

[92] Mohammed A.A., Rahim A.A.F. (2020). Experimental Behavior and Analysis of High Strength Concrete Beams Reinforced with PET Waste Fiber. Constr. Build. Mater..

[93] Sabireen, Butt F., Ahmad A., Ullah K., Zaid O., Ahmed Shah H., Kamal T. (2023). Mechanical Performance of Fiber-Reinforced Concrete and Functionally Graded Concrete with Natural and Recycled Aggregates. Ain Shams Eng. J..

[94] Parhi S.K., Patro S.K. (2024). Application of R-Curve, ANCOVA, and RSM Techniques on Fracture Toughness Enhancement in PET Fiber-Reinforced Concrete. Constr. Build. Mater..

[95] Al-Tulaian B.S., Al-Shannag M.J., Al-Hozaimy A.R. (2016). Recycled Plastic Waste Fibers for Reinforcing Portland Cement Mortar. Constr. Build. Mater..

[96] Peng Q., Chen B., Lu Q., Li K., Jin W. (2023). Effect of Steel-Waste PET Hybrid Fiber on Properties of Recycled Aggregate Concrete Based on Response Surface Methodology. Constr. Build. Mater..

[97] Signorini C., Volpini V. (2021). Mechanical Performance of Fiber Reinforced Cement Composites Including Fully-Recycled Plastic Fibers. Fibers.

[98] Mistry M., Rane G. (2021). Effect of PET Bottle Pieces and Waste Wrapper Fibers on Concrete Compressive Strength. IOP Conf. Ser. Mater. Sci. Eng..

[99] Lazorenko G., Kasprzhitskii A., Fini E.H. (2022). Sustainable Construction via Novel Geopolymer Composites Incorporating Waste Plastic of Different Sizes and Shapes. Constr. Build. Mater..

[100] Ghrieb A., Abadou Y., Choungara T., Bustamante R. (2022). Physical-Mechanical Evaluation of Polyethylene Terephthalate Fiber Dune Sand Mortar Exposed to Elevated Temperature. Sel. Sci. Pap. J. Civ. Eng..

[101] Marthong C., Sarma D.K. (2016). Influence of PET Fiber Geometry on the Mechanical Properties of Concrete: An Experimental Investigation. Eur. J. Environ. Civ. Eng..

[102] Prabhu P.G., Kumar C.A., Pandiyaraj R., Rajesh P., Kumar L.S. (2014). Impact Factor(JCC): 1.5548-Study on utilization of waste PET bottle fiber in concrete. Dimension.

[103] Salhotra S., Khitoliya R.K., Kumar S. (2021). Comparative Study of Uncoated and Coated Waste PET Fiber for Sustainable Concrete. Mater. Today Proc..

[104] Meza A., Siddique S. (2019). Effect of Aspect Ratio and Dosage on the Flexural Response of FRC with Recycled Fiber. Constr. Build. Mater..

[105] Bayraktar O.Y., Kaplan G., Shi J., Benli A., Bodur B., Turkoglu M. (2023). The Effect of Steel Fiber Aspect-Ratio and Content on the Fresh, Flexural, and Mechanical Performance of Concrete Made with Recycled Fine Aggregate. Constr. Build. Mater..

[106] Rasheed L.S., Alyhya W.S., Kadhim S.K. (2021). Utilising PET Bottle Fibers in the Production of Concrete. Proc. J. Phys. Conf. Ser..

[107] Rostami R., Zarrebini M., Mandegari M., Mostofinejad D., Abtahi S.M. (2020). A Review on Performance of Polyester Fibers in Alkaline and Cementitious Composites Environments. Constr. Build. Mater..

[108] Bhogle C.S., Pandit A.B. (2018). Ultrasound-Assisted Alkaline Hydrolysis of Waste Poly (Ethylene Terephthalate) in Aqueous and Non-Aqueous Media at Low Temperature. Indian. Chem. Eng..

[109] Alani A.M., Beckett D. (2013). Mechanical Properties of a Large Scale Synthetic Fibre Reinforced Concrete Ground Slab. Constr. Build. Mater..

[110] Lin X., Yu J., Li H., Lam J.Y.K., Shih K., Sham I.M.L., Leung C.K.Y. (2018). Recycling Polyethylene Terephthalate Wastes as Short Fibers in Strain-Hardening Cementitious Composites (SHCC). J. Hazard. Mater..

[111] Arias J.J.R., Thielemans W. (2021). Instantaneous Hydrolysis of PET Bottles: An Efficient Pathway for the Chemical Recycling of Condensation Polymers. Green. Chem..

[112] Chaduvula U., Viswanadham B.V.S., Kodikara J. (2017). A Study on Desiccation Cracking Behavior of Polyester Fiber-Reinforced Expansive Clay. Appl. Clay Sci..

[113] Oh R.-O., Ryu Y.-S., Park C.-G., Park S.-K. (2023). A Study on Performance Evaluation of Fiber Reinforced Concrete Using PET Fiber Reinforcement. J. Korean Tunn. Undergr. Space Assoc..

[114] Thomas L.M., Moosvi S.A. (2020). Hardened Properties of Binary Cement Concrete with Recycled PET Bottle Fiber: An Experimental Study. Mater. Today Proc..

[115] Meena A., Surendranath A., Ramana P. (2022). V Assessment of Mechanical Properties and Workability for Polyethylene Terephthalate Fiber Reinforced Concrete. Mater. Today Proc..

[116] da Luz Garcia M., Oliveira M.R., Silva T.N., Castro A.C.M. (2021). Performance of Mortars with PET. J. Mater. Cycles Waste Manag..

[117] Saxena R., Gupta T., Sharma R.K., Chaudhary S., Jain A. (2020). Assessment of Mechanical and Durability Properties of Concrete Containing PET Waste. Sci. Iran..

[118] Bamigboye G.O., Tarverdi K., Umoren A., Bassey D.E., Okorie U., Adediran J. (2021). Evaluation of Eco-Friendly Concrete Having Waste PET as Fine Aggregates. Clean. Mater..

[119] Guendouz M., Debieb F., Boukendakdji O., Kadri E.H., Bentchikou M., Soualhi H. (2016). Use of Plastic Waste in Sand Concrete. J. Mater. Environ. Sci..

[120] Kassa R.B., Kanali C., Ambassah N. (2019). Engineering Properties of Polyethylene Terephthalate Fibre Reinforced Concrete with Fly Ash as a Partial Cement Replacement. Civ. Environ. Res..

[121] Almeshal I., Tayeh B.A., Alyousef R., Alabduljabbar H., Mohamed A.M. (2020). Eco-Friendly Concrete Containing Recycled Plastic as Partial Replacement for Sand. J. Mater. Res. Technol..

[122] Górak P., Postawa P., Natalia Trusilewicz L., Łagosz A. (2021). Lightweight PET Based Composite Aggregates in Portland Cement Materials-Microstructure and Physicochemical Performance. J. Build. Eng..

[123] Jacob-Vaillancourt C., Sorelli L. (2018). Characterization of Concrete Composites with Recycled Plastic Aggregates from Postconsumer Material Streams. Constr. Build. Mater..

[124] Arjomandi A., Nematzadeh M., Fakoor M. (2023). Effect of bar yielding and heat on bond behavior between steel bar and high-strength concrete containing waste PET by pullout and beam tests: Experiments and predictions. Constr. Build. Mater..

[125] Iucolano F., Liguori B., Caputo D., Colangelo F., Cioffi R. (2013). Recycled Plastic Aggregate in Mortars Composition: Effect on Physical and Mechanical Properties. Mater. Des..

[126] Wiliński D., Łukowski P., Rokicki G. (2016). Application of Fibres from Recycled PET Bottles for Concrete Reinforcement. J. Build. Chem..

[127] Azhdarpour A.M., Nikoudel M.R., Taheri M. (2016). The Effect of Using Polyethylene Terephthalate Particles on Physical and Strength-Related Properties of Concrete; A Laboratory Evaluation. Constr. Build. Mater..

[128] Akinyele J.O., Ajede A. (2018). The Use of Granulated Plastic Waste in Structural Concrete. Afr. J. Sci. Technol. Innov. Dev..

[129] Colangelo F., Cioffi R., Liguori B., Iucolano F. (2016). Recycled Polyolefins Waste as Aggregates for Lightweight Concrete. Compos. B Eng..

[130] Abu-Saleem M., Zhuge Y., Hassanli R., Ellis M., Rahman M., Levett P. (2021). Evaluation of Concrete Performance with Different Types of Recycled Plastic Waste for Kerb Application. Constr. Build. Mater..

[131] Lerna M., Foti D., Petrella A., Sabbà M.F., Mansour S. (2023). Effect of the Chemical and Mechanical Recycling of PET on the Thermal and Mechanical Response of Mortars and Premixed Screeds. Materials.

[132] Záleská M., Pavlíková M., Pokorný J., Jankovský O., Pavlík Z., Černý R. (2018). Structural, Mechanical and Hygrothermal Properties of Lightweight Concrete Based on the Application of Waste Plastics. Constr. Build. Mater..

[133] Kaur G., Pavia S. (2021). Chemically Treated Plastic Aggregates for Eco-Friendly Cement Mortars. J. Mater. Cycles Waste Manag..

[134] Shubbar S.D.A., Al-Shadeedi A.S. (2017). Utilization of Waste Plastic Bottles as Fine Aggregate in Concrete. Kufa J. Eng..

[135] Alshkane Y., Boiny H.U., Alshkane Y.M., Rafiq S.K. Mechanical Properties of Cement Mortar by Using Polyethylene Terephthalate Fibers. Proceedings of the 5th National and 1st International Conference on Modern Materials and Structures in Civil Engineering.

[136] Pachta V., Triantafyllaki S., Stefanidou M. (2018). Performance of Lime-Based Mortars at Elevated Temperatures. Constr. Build. Mater..

[137] Armando L.S., Pereiro-Barceló J., Foti D., Ivorra S. (2024). Detailed Numerical Micro-modelling of Masonry TRM Reinforcements. International Conference on Structural Analysis of Historical Constructions.

[138] Nibudey R.N., Nagarnaik P.B., Parbat D.K., Pande A.M. (2014). Compressive Strength and Sorptivity Properties of Pet Fiber Reinforced Concrete. Int. J. Adv. Eng. Technol..

[139] Ge Z., Yue H., Sun R. (2015). Properties of Mortar Produced with Recycled Clay Brick Aggregate and PET. Constr. Build. Mater..

[140] Azmi N.B., Khalid F.S., Irwan J.M., Mazenan P.N., Zahir Z., Shahidan S. (2018). Performance of Composite Sand Cement Brick Containing Recycle Concrete Aggregate and Waste Polyethylene Terephthalate with Different Mix Design Ratio. Proc. IOP Conf. Ser. Earth Environ..

[141] Kurdowski W. (2014). Cement and Concrete Chemistry.

[142] Chinchillas-Chinchillas M.J., Gaxiola A., Alvarado-Beltrán C.G., Orozco-Carmona V.M., Pellegrini-Cervantes M.J., Rodríguez-Rodríguez M., Castro-Beltrán A. (2020). A New Application of Recycled-PET/PAN Composite Nanofibers to Cement–Based Materials. J. Clean. Prod..

[143] Liu Z. (2015). Experimental Research on the Engineering Characteristics of Polyester Fiber–Reinforced Cement-Stabilized Macadam. J. Mater. Civ. Eng..

[144] Onuaguluchi O., Banthia N. (2017). Durability Performance of Polymeric Scrap Tire Fibers and Its Reinforced Cement Mortar. Mater. Struct./Mater. Constr..

[145] Koo B.M., Kim J.H.J., Kim S.B., Mun S. (2014). Material and Structural Performance Evaluations of Hwangtoh Admixtures and Recycled PET Fiber-Added Eco-Friendly Concrete for CO_2_ Emission Reduction. Materials.

[146] Mazzoli A., Monosi S., Plescia E.S. (2015). Evaluation of the Early-Age-Shrinkage of Fiber Reinforced Concrete (FRC) Using Image Analysis Methods. Constr. Build. Mater..

[147] Yilmaz A. (2021). Mechanical and Durability Properties of Cement Mortar Containing Waste Pet Aggregate and Natural Zeolite. J. Ceram. Silik..

[148] Revathi S., Suresh D., Anwar S.T. (2023). Behaviour of Concrete with PET Bottles as Fibers & Silica Fume as Partial Replacement of Cement. Mater. Today Proc..

[149] Noroozi R., Shafabakhsh G., Kheyroddin A., Mohammadzadeh Moghaddam A. (2019). Investigating the Effects of Recycled PET Particles, Shredded Recycled Steel Fibers and Metakaolin Powder on the Properties of RCCP. Constr. Build. Mater..

[150] Bui N.K., Satomi T., Takahashi H. (2018). Recycling Woven Plastic Sack Waste and PET Bottle Waste as Fiber in Recycled Aggregate Concrete: An Experimental Study. Waste Manag..

[151] Tafheem Z., Rakib R.I., Esharuhullah M.D., Alam S.R., Islam M.M. (2018). Experimental investigation on the properties of concrete containing post-consumer plastic waste as coarse aggregate replacement. J. Mater. Eng. Struct..

[152] Singh K. (2020). Partial Replacement of Cement with Polyethylene Terephthalate Fiber to Study Its Effect on Various Properties of Concrete. Proc. Mater. Today Proc..

[153] Ávila Córdoba L., Martínez-Barrera G., Barrera Díaz C., Ureña Nuñez F., Loza Yañez A. (2013). Effects on Mechanical Properties of Recycled PET in Cement-Based Composites. Int. J. Polym. Sci..

[154] Umasabor R.I., Daniel S.C. (2020). The Effect of Using Polyethylene Terephthalate as an Additive on the Flexural and Compressive Strength of Concrete. Heliyon.

[155] Rathore R.S., Prakash D., Chouhan H.S. (2024). Mechanical and Microstructural Analysis of Cement Mortar Mixes Using PET Plastic Waste. Proc. IOP Conf. Ser. Earth Environ. Sci..

[156] Dawood A.O., AL-Khazraji H., Falih R.S. (2021). Physical and Mechanical Properties of Concrete Containing PET Wastes as a Partial Replacement for Fine Aggregates. Case Stud. Constr. Mater..

[157] Kangavar M.E., Lokuge W., Manalo A., Karunasena W., Frigione M. (2022). Investigation on the Properties of Concrete with Recycled Polyethylene Terephthalate (PET) Granules as Fine Aggregate Replacement. Case Stud. Constr. Mater..

[158] Shamsudin M.M.H., Hamid N.H., Fauzi M.A.M. (2021). Compressive and Flexural Strength of Concrete Containing Recycled Polyethylene Terephthalate (PET). Key Eng. Mater..

[159] Nkomo N.Z., Masu L.M., Nziu P.K. (2022). Effects of Polyethylene Terephthalate Fibre Reinforcement on Mechanical Properties of Concrete. Adv. Mater. Sci. Eng..

[160] Amran M., Fediuk R., Abdelgader H.S., Murali G., Ozbakkaloglu T., Lee Y.H., Lee Y.Y. (2022). Fiber-Reinforced Alkali-Activated Concrete: A Review. J. Build. Eng..

[161] Çolak A.B., Akçaözoğlu K., Akçaözoğlu S., Beller G. (2021). Artificial Intelligence Approach in Predicting the Effect of Elevated Temperature on the Mechanical Properties of PET Aggregate Mortars: An Experimental Study. Arab. J. Sci. Eng..

[162] Silva E.R., Coelho J.F.J., Bordado J.C. (2013). Strength Improvement of Mortar Composites Reinforced with Newly Hybrid-Blended Fibres: Influence of Fibres Geometry and Morphology. Constr. Build. Mater..

[163] Foti D. (2016). Innovative Techniques for Concrete Reinforcement with Polymers. Constr. Build. Mater..

[164] Foti D. (2013). Use of Recycled Waste Pet Bottles Fibers for the Reinforcement of Concrete. Compos. Struct..

[165] Adnan H.M., Dawood A.O. (2020). Strength Behavior of Reinforced Concrete Beam Using Re-Cycle of PET Wastes as Synthetic Fibers. Case Stud. Constr. Mater..

[166] Silva D.A., Betioli A.M., Gleize P.J.P., Roman H.R., Gómez L.A., Ribeiro J.L.D. (2005). Degradation of Recycled PET Fibers in Portland Cement-Based Materials. Cem. Concr. Res..

[167] Al-Hadithi A.I., Noaman A.T., Mosleh W.K. (2019). Mechanical Properties and Impact Behavior of PET Fiber Reinforced Self-Compacting Concrete (SCC). Compos. Struct..

[168] Asdollah-Tabar M., Heidari-Rarani M., Aliha M.R.M. (2021). The Effect of Recycled PET Bottles on the Fracture Toughness of Polymer Concrete. Compos. Commun..

[169] Belmokaddem M., Mahi A., Senhadji Y., Pekmezci B.Y. (2020). Mechanical and Physical Properties and Morphology of Concrete Containing Plastic Waste as Aggregate. Constr. Build. Mater..

[170] Lu C., Leung C.K.Y. (2017). Effect of Fiber Content Variation on the Strength of the Weakest Section in Strain Hardening Cementitious Composites (SHCC). Constr. Build. Mater..

[171] Pan Z., Wu C., Liu J., Wang W., Liu J. (2015). Study on Mechanical Properties of Cost-Effective Polyvinyl Alcohol Engineered Cementitious Composites (PVA-ECC). Constr. Build. Mater..

[172] Ding C., Guo L.P., Chen B. (2019). Theoretical Analysis on Optimal Fiber-Matrix Interfacial Bonding and Corresponding Fiber Rupture Effect for High Ductility Cementitious Composites. Constr. Build. Mater..

[173] Foti D., Paparella F. (2014). Impact Behavior of Structural Elements in Concrete Reinforced with PET Grids. Mech. Res. Commun..

[174] Hama S.M. (2022). Behavior of Concrete Incorporating Waste Plastic as Fine Aggregate Subjected to Compression, Impact Load and Bond Resistance. Eur. J. Environ. Civ. Eng..

